# Paradoxical sex-specific patterns of autoantibody response to SARS-CoV-2 infection

**DOI:** 10.1186/s12967-021-03184-8

**Published:** 2021-12-30

**Authors:** Yunxian Liu, Joseph E. Ebinger, Rowann Mostafa, Petra Budde, Jana Gajewski, Brian Walker, Sandy Joung, Min Wu, Manuel Bräutigam, Franziska Hesping, Elena Rupieper, Ann-Sophie Schubert, Hans-Dieter Zucht, Jonathan Braun, Gil Y. Melmed, Kimia Sobhani, Moshe Arditi, Jennifer E. Van Eyk, Susan Cheng, Justyna Fert-Bober

**Affiliations:** 1 Department of Cardiology, Smidt Heart Institute, Cedars-Sinai Medical Center, Los Angeles, CA USA; 2 Advanced Clinical Biosystems Institute, Department of Biomedical Sciences, Cedars-Sinai Medical Center, Los Angeles, CA USA; 3 Division of Gastroenterology, Department of Medicine, Cedars-Sinai Medical Center, Los Angeles, CA USA; 4 Department of Pathology and Laboratory Medicine, Cedars-Sinai Medical Center, Los Angeles, CA USA; 5 Oncimmune Germany GmbH, Dortmund, Germany; 6 Department of Pediatrics, Division of Infectious Diseases and Immunology, Infectious and Immunologic Diseases Research Center (IIDRC), and Department of Biomedical Sciences, Cedars-Sinai Medical Center, Los Angeles, CA USA

**Keywords:** SARS-CoV-2, COVID-19, AABs, Autoantigen selectivity, Sex differences

## Abstract

**Background:**

Pronounced sex differences in the susceptibility and response to SARS-CoV-2 infection remain poorly understood. Emerging evidence has highlighted the potential importance of autoimmune activation in modulating the acute response and recovery trajectories following SARS-CoV-2 exposure. Given that immune-inflammatory activity can be sex-biased in the setting of severe COVID-19 illness, the aim of the study was to examine sex-specific autoimmune reactivity to SARS-CoV-2 in the absence of extreme clinical disease.

**Methods:**

In this study, we assessed autoantibody (AAB) reactivity to 91 autoantigens previously linked to a range of classic autoimmune diseases in a cohort of 177 participants (65% women, 35% men, mean age of 35) with confirmed evidence of prior SARS-CoV-2 infection based on presence of antibody to the nucleocapsid protein of SARS-CoV-2. Data were compared to 53 pre-pandemic healthy controls (49% women, 51% men). For each participant, socio-demographic data, serological analyses, SARS-CoV-2 infection status and COVID-19 related symptoms were collected by  an electronic survey of questions. The symptoms burden score was constructed based on the total number of reported symptoms (N = 21) experienced within 6 months prior to the blood draw, wherein a greater number of symptoms corresponded to a higher score and assigned as more severe burden.

**Results:**

In multivariable analyses, we observed sex-specific patterns of autoreactivity associated with the presence or absence (as well as timing and clustering of symptoms) associated with prior COVID-19 illness. Whereas the overall AAB response was more prominent in women following asymptomatic infection, the breadth and extent of AAB reactivity was more prominent in men following at least mildly symptomatic infection. Notably, the observed reactivity included distinct antigens with molecular homology with SARS-CoV-2.

**Conclusion:**

Our results reveal that prior SARS-CoV-2 infection, even in the absence of severe clinical disease, can lead to a broad AAB response that exhibits sex-specific patterns of prevalence and antigen selectivity. Further understanding of the nature of triggered AAB activation among men and women exposed to SARS-CoV-2 will be essential for developing effective interventions against immune-mediated sequelae of COVID-19.

**Supplementary Information:**

The online version contains supplementary material available at 10.1186/s12967-021-03184-8.

## Introduction

Mechanisms underlying sex differences in both susceptibility and response to SARS-CoV-2 infection remain poorly understood. Biological sex differences have become manifest with respect to vulnerability to infection, adaptive immune responses, and the equilibrium of inflammation and tissue repair in the resolution of infection [1]. Recent evidence points to the possible contributions of triggering and persistence of autoimmune activation in SARS-CoV-2-infected COVID-19 patients [2, 3]. Intriguingly, despite classic autoimmune diseases being more prevalent in females, emerging studies have revealed a paradoxical male predominance of autoimmune activation in the setting of severe COVID-19 illness [4]. The extent to which such paradoxical sex differences in triggered autoimmunity may exist and persist across the broader clinical spectrum of SARS-CoV-2 infection is unclear. Recognizing that sex bias is potentially introduced when assessing autoimmune activation in the setting of more severe forms of COVID-19 illness, we deliberately aimed to interrogate sex-specific autoimmune activation after SARS-CoV-2 exposure in the absence of any extreme manifestations of clinical disease. Therefore, using an array to detect autoantibodies (AABs) to 91 antigens previously linked to a range of classic autoimmune diseases, we sought to comprehensively examine the diversity of AAB responses in male and female health care workers (HCWs) who were exposed to SARS-CoV-2 and self-reported presence or absence of distinct symptoms associated with COVID-19. Importantly retrospective symptoms were correlated with autoimmune response to ascertain potential prevalence of long-lasting symptoms driven by host autoimmune response.

## Methods

### Study sample

Our primary study cohort was derived from a diverse and unselected population of adults employed in a multisite healthcare delivery system located in Los Angeles County, including individuals with direct patient contact and others with non-patient-oriented work functions. A total of N = 6062 individuals (age 41 ± 12 years, 68% women) had complete data from both the serology testing and symptoms survey. Of those individuals who completed a full survey of symptoms potentially related to COVID-19 illness in the preceding 6 months (N = 3688), a total of 177 (5%) tested positive for anti-nucleocapsid IgG serology results (Abbott Diagnostics, Abbott Park, Illinois) (64% women, 36% men) [5]. Of note, 23 (13%) of the seropositive individuals reported absence of prior symptoms (i.e. asymptomatic status). The positive anti-nucleocapsid IgG serology results were reported as a signal to cut-off (S/CO) ratio corresponding to the relative light units produced by the test sample compared with the relative light units produced by an assay calibrator sample. The manufacturer-recommended S/CO ratio of 1.4 was used to assign binary seropositivity status [5–7].

All study protocols were approved by the Cedars-Sinai institutional review board and all participants provided written informed consent. Our secondary cohorts included healthy controls (HCs) (N = 53, 49% women, 51% men) who provided pre-pandemic serum samples obtained from the Bavarian Red Cross (Wiesentheid, Germany) with ethical approval from the Bayerische Landesaerztekammer (Study No. 01/09). We also included a classic autoimmune disease comparator cohort (N = 6, 83% women, 17% men) of systemic lupus erythematosus (SLE) patients who fulfilled the American College of Rheumatology (ACR) classification criteria for SLE [8] and had pre-pandemic serum samples collected by BioIVT (West Sussex, United Kingdom).

### Autoantibody assays

We utilized a panel of 91 protein antigens and cytokines in a multiplexed bead-based assay using Luminex FlexMAP 3D technology (Additional file 1: Table S1) [9]. The selected analytical targets can be grouped into different functional protein families or belong to immune-relevant biological pathways [10]. Antigens were covalently coupled to distinct carboxylated magnetic beads (Luminex Corp, Austin, Texas) by carbodiimide as per previously developed antigen-coupling protocols [10]. Following technical recommendations from Luminex, we titrated within the 1 to 125 μg range to determine the optimal amount of protein (up to 97 μg, and average 30 μg) to achieve a specific coupling reaction when applied to resuspended microspheres (5.0 × 10^6^ MagPlex™ beads). Coupling efficiency was confirmed by incubation of 625 beads from each coupled region with a phycoerythrin-conjugated anti-6 × HisTag antibody (Abcam, Cambridge, UK) at a concentration of 10 μg/mL for 45 min shaking at 900 rpm and room temperature. Coupled beads were mixed to a final concentration of 62.5 beads/μL and stored in PBS supplemented with 1% bovine serum albumin, 0.1% Tween 20 and 0.05% ProClin™ 300 (Merck KGaA, Darmstadt, Germany), at 4 °C until use [10]. For analysis, serum or plasma samples were diluted 1:100 in assay buffer (50% PBS with 1% BSA, 50% LowCross-Buffer® (Candor Biosciences, Wangen, Germany), 1.3 µg/µl *E. coli* lysate) and incubated for 20 min at room temperature. Next, the bead’s mix (50 μL) was added to each well of a 96-well plate and incubated with 50 μL of diluted sample (1:100) in assay buffer for 22 h at 4–8 °C in a plate shaker (900 rpm). Subsequently, after washing with PBS/0.1% Tween 20 the beads were incubated with R-phycoerythrin-labelled goat anti-human IgG detection antibody (Ab) (5 µg/mL, Dianova, Hamburg, Germany) for 1 h at RT and washed again. The beads were analyzed in a FlexMap3D instrument (Luminex Corporation, Austin, Texas). The IgG reactivity values were given as median fluorescence intensity (MFI) and antigens fulfilling the minimum bead count criterion (> 10 beads measured per bead ID) were exported for data analysis. In addition, triplicates of a COVID-19 positive reference sample (comprised of three HCWs) were run on each plate to calculate median intra-plate coefficient of variation (CV) and median inter-plate CV. The dynamic range was determined using blank samples and a control bead coupled to BSA (control_BSA) for the lower and huIgG (control_huIgGhi) for the upper MFI range. We standardized the log MFI value of each AAB across all of our study samples (including 177 HCW, 6 SLE, and 53 HC), by first subtracting the mean and then dividing by the standard deviation. Notwithstanding variation in samples sizes for positive (SLE) and negative (HC) controls, we additionally applied methods for normalizing values for both positive and negative controls.

### Statistical analyses

Parametric tests and non-parametric tests were used to compare normally distributed continuous variables and non-normally distributed or categorical variables, respectively. Histograms were used to display distribution of symptomatology as well as AABs reactivity against each antigen for the cohort in sex-pooled and sex-specific analyses. Original logistic regression was used to examine the associations between AABs reactivity of each antigen and self-reported symptoms burden, defined as a symptoms burden score. The symptoms burden score was constructed based on the total number of reported symptoms (specifically defined as: chest pain, chills, conjunctivitis, dry cough, productive cough, diarrhea, fatigue, fever, headache, loss of appetite, muscle aches, nasal congestion, nausea, runny nose, shortness of breath, skin, smell taste, sneezing, sore throat, stroke symptoms, vomiting) experienced at any time within 6 months prior to the blood draw, as detailed previously [5]. Presence of each symptom was assigned a score of 1 point, such that the greater number of symptoms experienced corresponded to a higher symptoms burden score. Total symptom burden was, in turn, classified into 3 categories: asymptomatic was represented by a score of 0, mild symptom burden as a score of 1 to 7, and more than mild symptom burden as a score of > 7. Additionally, we calculated a categorical score to represent symptom timing, using data collected in association with each reported symptom: no symptoms (symptom timing score = 0), symptoms within the prior 6 months (symptom timing score = 1), symptoms within the prior 3 months (symptom timing score = 2), symptoms within the prior 2 months (symptom timing score = 3), symptoms within the prior 1 month (symptom timing score = 4), and now, or within the last week (symptom timing score = 5). Prior to linear multivariable-adjusted analyses, AAB index (MFI) was log(2) transformed. In adjusted analyses, we compared differences between AABs response status in each variable of interest, grouped into one of three categories: (1) demographic characteristics (e.g., sex, age); (2) COVID-19-related response variables (e.g., self-reported fever, chills, dry cough, anosmia, nausea, and so on and timing of reported symptoms), and (3) SARS-CoV-2 IgG anti-nucleocapsid (N) antibody index (anti-N IgG index). In multivariable-adjusted analyses, we used linear models to examine the extent to which the three categories of variables (predictors) may be associated with AABs response. We also performed sex-specific clustering and co-occurrence network analyses to clarify similarities and differences between sexes in the AAB response to SARS-CoV-2 exposure. For clustering analyses, we first applied principal components analysis (PCA) reduction on the AAB expression and selected components that cumulatively explained at least 85% of variation in AAB response; we then optimized K-means clustering using the gap statistic to estimate the number as well as membership of clusters [11]. For co-occurrence analyses, we estimated and then visualized probabilistic pairwise co-occurrence of symptoms with final networks constructed based on sex-specific frequencies of co-occurrences [12]. All statistical analyses were conducted using R (v3.5.1) and statistical significance was defined as a two-tailed P value < 0.05.

## Results

The primary study cohort included HCW participants identified as prior SARS-CoV-2 infected individuals based on a positive anti-N IgG index. The comparator study cohorts included 53 HCs and 6 SLE patients. The demographic, clinical, exposure and symptom response characteristics of the study sample are shown in Additional file 1: Table S2. The primary cohort of N = 177 HCWs had a mean age of 35 [IQR: 30–44] years and included 65% women, 68% of non-white race, and 28% of Hispanic/Latinx ethnicity. For this cohort, we graded severity of prior SARS-CoV-2 infection based on reported symptoms burden (based on up to 21 distinct possible symptoms) and timing (0 to 6 months prior to assessment and assays). We categorized HCWs based on the number of reported symptoms with into three groups, asymptomatic with no symptoms reported (N = 23), mildly symptomatic with 1 to 7 symptoms reported (N = 64) and more than mildly symptomatic (N = 90) with more than 7 symptoms reported. Rates of sex-specific positivity for the AABs detected were not significantly different between the HCW cases and HC controls. Among the HCW cases, males demonstrated an overall broader and more diverse elevation in AAB levels than females, particularly in relation to greater symptom burden. Among asymptomatic individuals, AAB reactivity to 25 out of 91 antigens (28%) was detected in men, whereas AAB reactivity to 66 out of 91 antigens (72%) was reported in women. By contrast, among individuals with mild symptoms, 58 out of 91 antigens (64%) showed positive reactivity in men versus 33 out of 91 (36%) in women. Among individuals with more than mild symptoms (N = 90), AABs to 75 out of 91 antigens (82%) showed positive reactivity in men versus only 16 out of 91 antigens (18%) in women.

### Sex‐specific frequency of symptoms

**Fig. 1:**
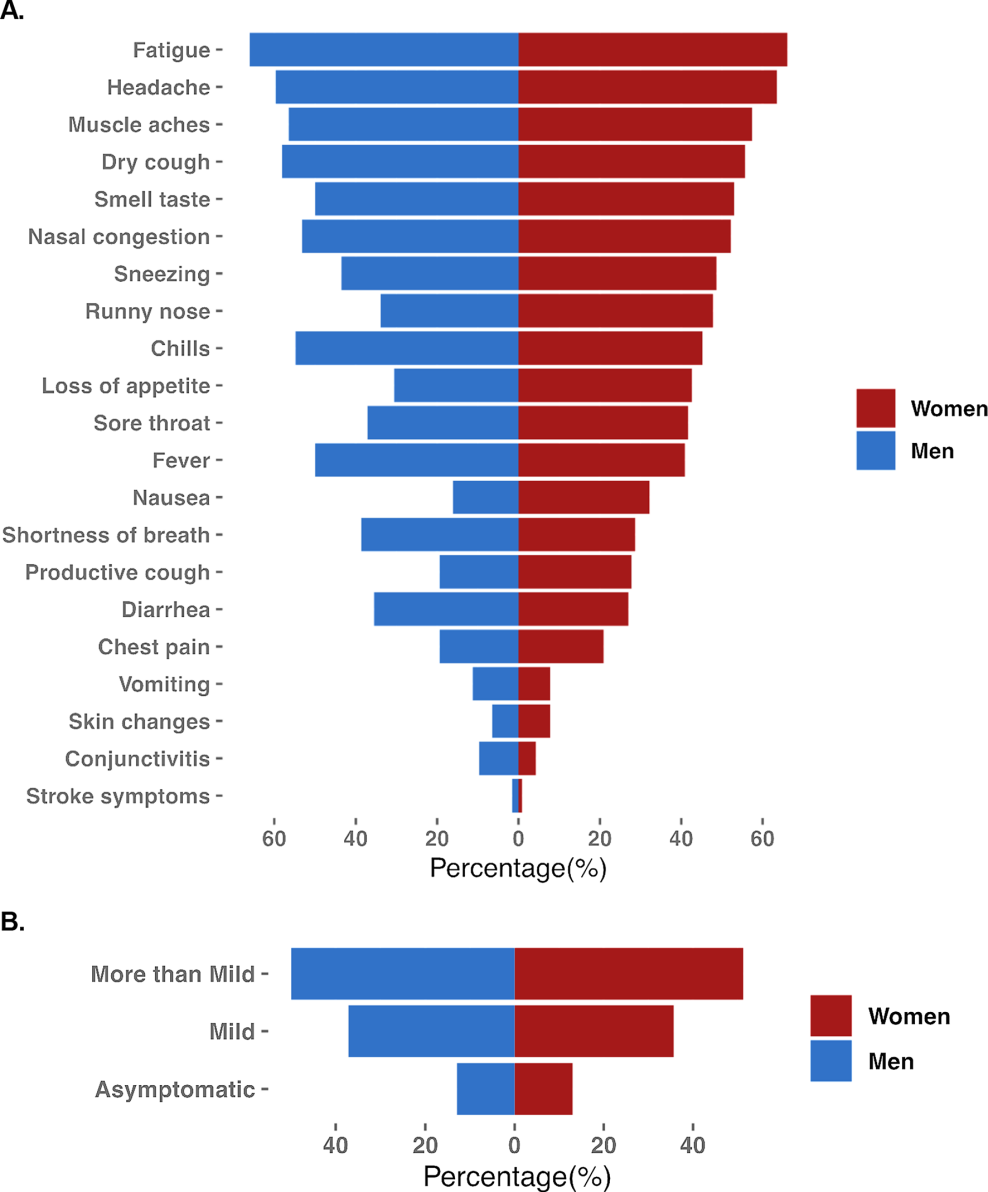
Sex-specific frequency of symptoms type and overall symptoms burden, in men and women previously infected by SARS-CoV-2. In our primary study cohort, the distribution of experienced symptoms was generally similar between men and women (**A**) with some exceptions including certain distinct symptoms being more frequent in men (e.g., chills, fever, conjunctivitis) and other distinct symptoms being more frequent in women (e.g., loss of appetite, nausea). In analyses of overall symptom burden, frequencies of asymptomatic, mildly symptomatic, and more than mildly symptomatic persons were relatively equally distributed between the sexes (**B**)

As shown in Fig. 1, the vast majority of symptoms related to prior SARS-CoV-2 infection were experienced similarly by men and women. Although specific symptoms appeared to be reported more frequently by men (e.g., chills, fever, shortness of breath, diarrhea, conjunctivitis) and other symptoms were reported more frequently by women (e.g., loss of appetite, nausea, and productive cough), these differences were not statistically significant (Additional file 1: Table S2). Similar to the specific types of symptoms assessed, we observed that varying degrees of total symptom burden were also distributed relatively equally between the sexes (Fig. 1). There was also no significant difference between females and males and timing of the reported symptoms. In co-occurrence analyses, we found among 21 total symptoms that co-occurrence of symptoms was significant for 137 symptom pairs in women compared to 91 symptom pairs in men (Additional file 1: Fig. S1). Notably the most frequently co-occurring symptoms in women (chest pain, productive cough, chills, headache, fever) were similar to those in men (chest pain, fever, headache, chills, nasal congestion, dry cough). Chest pain was the single most frequently co-occurring symptom in both sexes, and significantly paired with occurrence of 17 other symptoms in women and 10 other symptoms in men.

### Sex‐specific frequency of autoantibody response

**Fig. 2:**
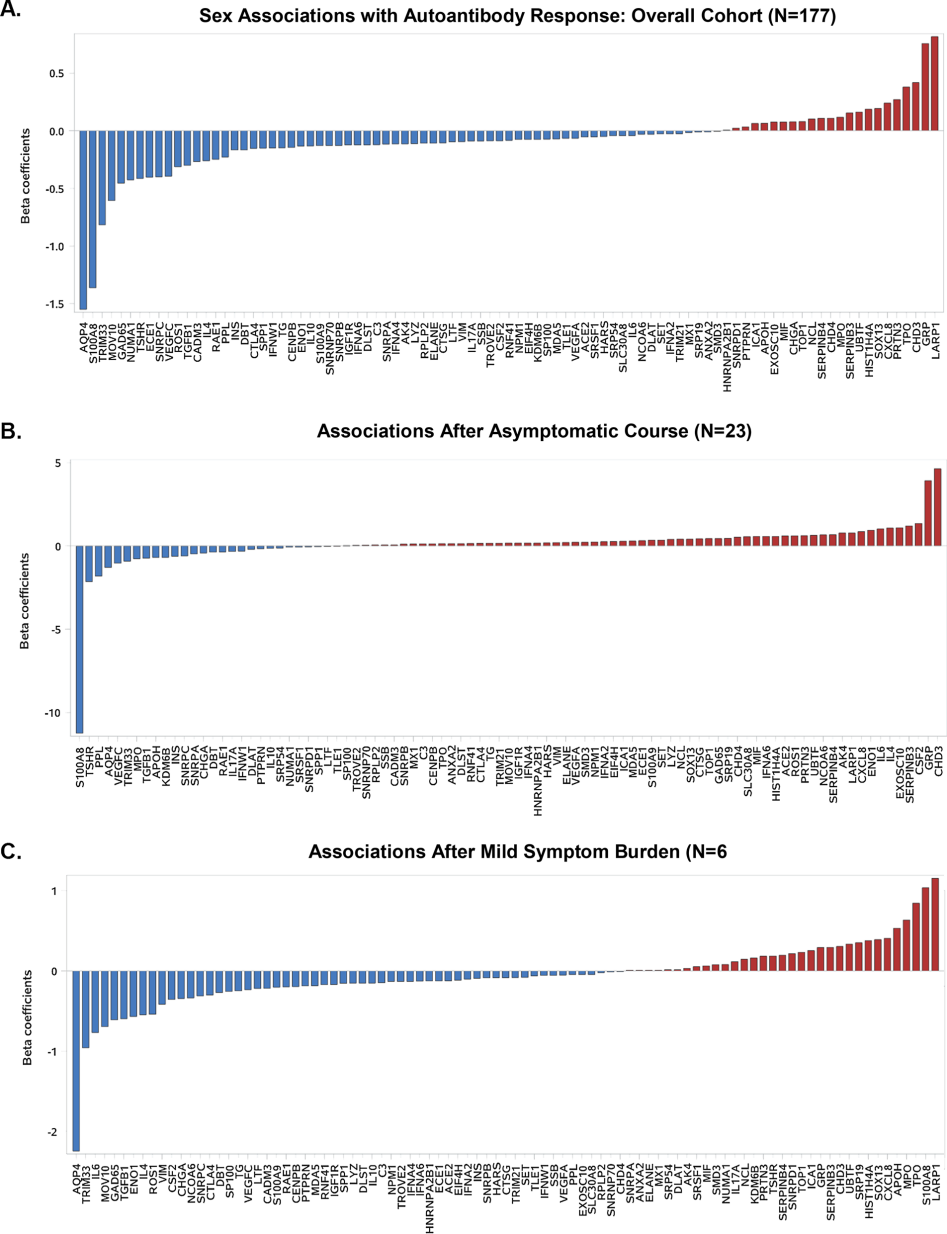

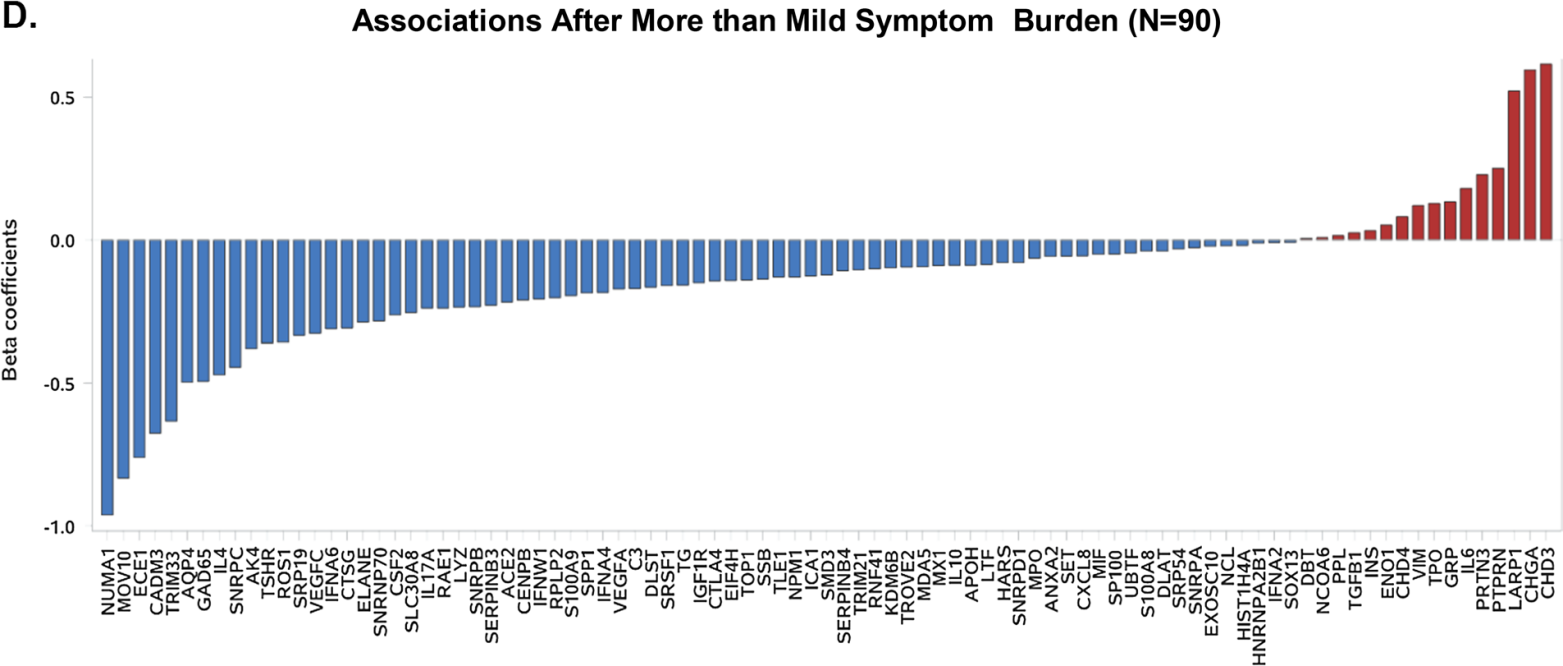
Sex associations with autoantibody activation by symptoms burden, in men and women previously infected by SARS-CoV-2. The age-adjusted associations of sex (female versus male) with AABs activation across the panel of 91 antigens are shown for the primary cohort overall (**A**) and within persons with varying loads of symptom burden: asymptomatic (**B**), mild symptom burden (**C**), more than mild symptom burden (**D**)

In age-adjusted regression analyses, we examined the association of sex (female versus male) with measured plasma levels for each of the 91 AABs assayed. Across the entire cohort, the majority of assayed AABs were associated with male sex and the minority with female sex but the sex-specific frequency and magnitudes of association varied by symptom burden (Fig. 2). Among asymptomatic individuals, the breadth and magnitude of AAB reactivity was much more pronounced in females compared to males (Fig. 2B). Notably, AABs to cytokine and chemokine antigens (IL6 and CSF2) involved in immune defense, together with lung specific proteins (gastrin release peptide (GRP) and serpin family B member 3 (SERPINB3), were predominantly elevated in asymptomatic females. By contrast, thyroid stimulating hormone receptor (TSHR) and lysine demethylase 6B (KDM6B), which are known primary antigens in autoimmune diseases, including Graves' disease and Hashimoto's disease [13] and systemic sclerosis (SS also termed scleroderma) [14] were highly expressed in asymptomatic males. Among all participants who had at least mild symptoms, the range and degree of AAB reactivity was more pronounced in males compared to females (Fig. 2C, D). The most abundant AABs in males were preferentially enriched in classical nuclear AABs such as small nuclear ribonucleoprotein polypeptide C (SNRPC) a target of autoreactive B cells and T cells in several rheumatic diseases including SLE, mixed connective tissue disease (MCTD) [15], inflammatory myositis, and SS [16]; nuclear mitotic apparatus protein 1 (NUMA1), antibody associated with SLE, SS, and RA [17]; and autoantibodies recognizing dihydrolipoamide succinyltransferase (DLST) found in primary biliary cirrhosis [18]. In this group, the highest expressed antibody was Moloney leukemia virus 10 homolog (MOV10), a putative RNA which inhibits replication of a number of retroviruses [19, 20] and recently showed to interacts with SARS-CoV-2 proteins [21].

In analyses repeated using AAB values normalized for both positive and negative controls, results were unchanged with primary sex-specific antigens consistently identified across the pre-specified disease severity groups including asymptomatic (i.e. TSHR and PPL in males; GRP, CSF2, and SERNIPB3 in females), mildly symptomatic (i.e. IL6, TRIM33, and MOV10 in males; LARP1, TPO, and MPO in females), and more than mildly symptomatic (i.e. MOV10, ECE1, and NUMA1 in males; LARP1 and PTPRN in females).

In sex-specific cluster analyses, we observed an overall broader distribution as well as larger number of distinct clusters of AABs in men compared to women particularly among the asymptomatic and mildly symptomatic groups (Additional file 1: Figs. S2 and S3). We also observed findings consistent with broader activation of AABs corresponding with greater symptom burden, a marker of relative illness severity, in both sexes (Additional file 1: Table S3).

### Sex-specific associations of autoantibody reactivity with symptoms

**Fig. 3:**
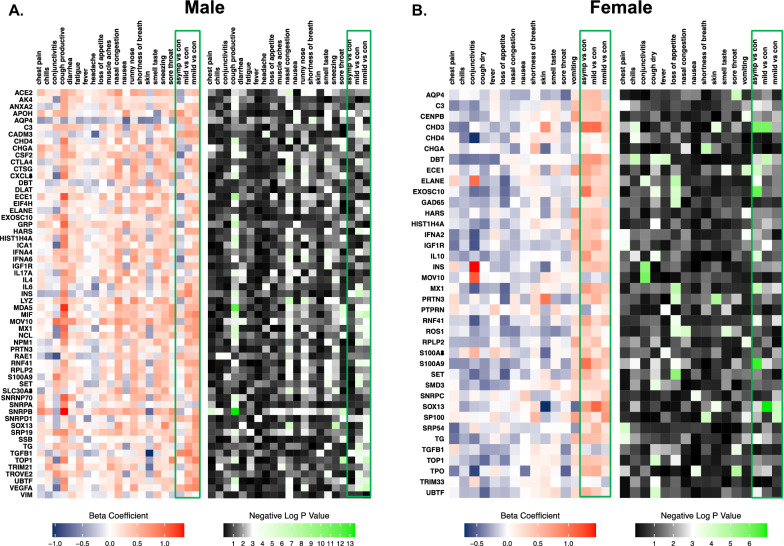
Sex-specific associations of autoantibody reactivity with individual symptoms, in persons previously infected by SARS-CoV-2. From age-adjusted regression analyses, beta coefficients and negative log p values were obtained from examining the associations of symptoms with distinct autoantibodies. Associations for men are shown in **A**. Associations for women are shown in **B**

**Fig. 4:**
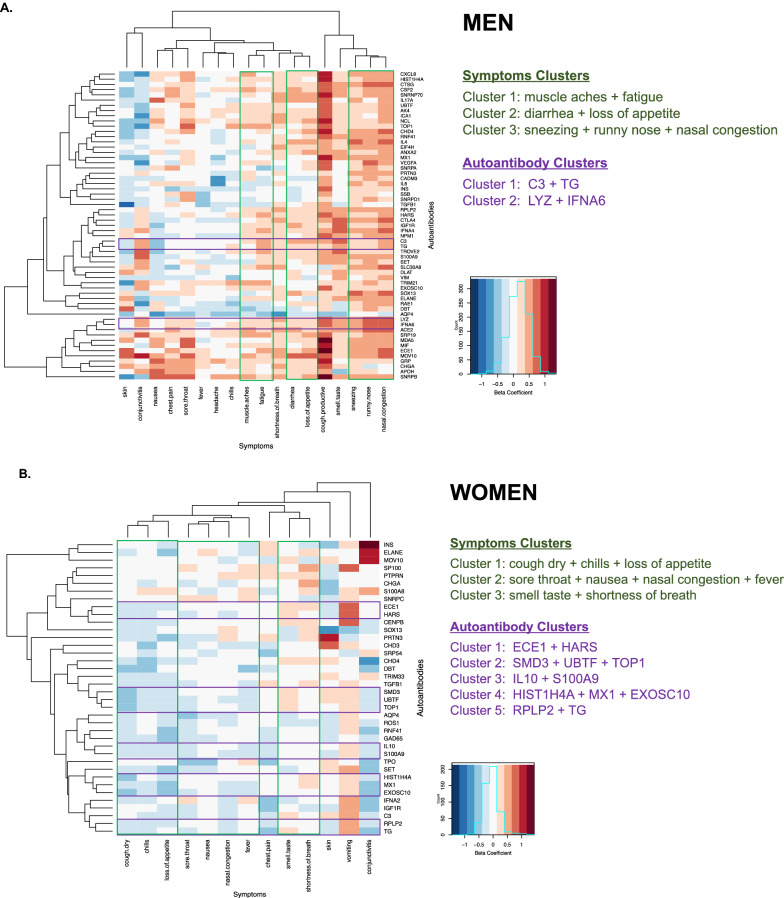
Sex-specific clustering of symptoms and autoantibodies in men and women previously infected with SARS-CoV-2. Symptoms and autoantibodies were grouped based on similar directions and magnitudes of the beta coefficients from age-adjusted regression association analyses, with clusters selected based on a threshold of h = 0.5 for autoantibodies and h = 1.5 for symptoms from Ward hierarchical clustering. Results are shown for men in (**a**) and women in (**b**)

In age-adjusted regression analyses we examined the sex-specific associations of distinct AABs levels with symptomatology (one symptom and AAB per model), as shown in Figs. 3 and 4, Additional file 1: Tables S4–S7). In unadjusted regression analyses, 42 of 91 autoantibodies showed statistically significant reactivity that correspond to 18 distinct symptoms for the whole cohort. In males, 63 out of 91 autoantibodies showed statistically significant reactivity corresponding to 18 distinct symptoms. In females, 41 out of 91 autoantibodies showed statistically significant reactivity corresponding to 14 symptoms. After age-adjusted regression analyses, among males, 59 out of 63 AABs were associated with 18 out of all 18 symptoms that demonstrated significant associations. Among females, 38 out of 41 AABs demonstrated significant beta coefficients in relation to 13 out of 14 symptoms (Fig. 3). Notably, in males, a large number of AABs had increased levels in relation to at least a mild overall symptoms burden while a substantial proportion of these same AABs exhibited lower levels in relation to asymptomatic status. By contrast, a smaller number of AABs exhibited generally consistently increased levels in relation to any level of overall symptom burden in females, including asymptomatic status. Results of analyzing response of distinct AABs in relation to timing of symptoms demonstrated similar sex-specificity (Additional file 1: Fig. S4). Notably, expression of ACE2, CSF2, LYZ, MDA5, MOV10, SNRPB, SOX13 and SRP19 autoantibodies appeared associated with overall timing score (representing a greater number of more recently experienced symptoms), indicating that these AABs were triggered and correlated with symptoms that were reported closer to timing of the AAB assay (P < 0.05 for all).

In males, among the significantly associated AABs, small nuclear ribonucleoprotein polypeptides B (SNRPB), chromodomain helicase DNA binding protein 4 (CHD4) and chromogranin A (CHGA) were the most frequently associated with the distinct symptoms of productive cough and nasal congestion. In females, the AABs to dihydrolipoamide branched chain transacylase E2 (DBT) and ROS proto-oncogene 1, receptor tyrosine kinase (ROS1), were the most frequently elevated in relation to dry cough and loss of appetite. Overall profiles of AAB reactivity in relation to symptoms indicated the most frequent and significantly associated AABs in males appeared to follow an SLE-related pattern (Additional file 1: Fig. S5), whereas AAB profiles in females were more suggestive of poly-autoimmunity [22].

To identify putative AAB sub-groups associated with potential discriminatory symptoms, we used hierarchical clustering analyses to identify similar magnitudes and directions of associations across AABs. The results of sex-specific two-dimensional clustering of symptoms variables in relation to AABs are shown in Fig. 4. In males, the initially identified cluster included the symptoms of muscle aches and fatigue (cluster 1), with diarrhea and loss of appetite clustered next (cluster 2), and sneezing, runny nose, and nasal congestion also clustered together (cluster 3). In females, there were also three major clusters identified: dry cough, chilis, and loss of appetite (cluster 1); sore throat, nausea, nasal congestion, and fever (cluster 2); and smell/taste change and shortness of breath (cluster 3). In males, clustering of AABs including C3 with TG antigens (cluster 1) and AABs to antigens representing LYZ and IFNA6 protein (cluster 2). In females, we found 5 clusters in total: ECE1 and HARS (cluster 1); SMD3, UBTF, and TOP1 (cluster 2); IL10 and S100A9 (cluster 3); HIST1H4A, MX1, and EXOSC10 (cluster 4); and RPLP2 and TG (cluster 5).

### Sex-specific associations of autoantibody reactivity and anti-N IgG levels

There was no significant difference between male and female participants in levels of anti-N IgG index (4.68 ± 2.02 vs 4.71 ± 2.07). In analyses of potential correlations between anti-N IgG index and AAB response, crude unadjusted correlations were seen for only 9 out of 91 autoantibodies: including APOH, MDA5, AK4, TSHR, MIF, SERPINB3, CENPB, TPO, MPO were reported to correlate with anti-N IgG index. However, in multivariable analyses adjusting for sex and age, all associations were non-significant. In sex-stratified analyses, putative associations between autoantibodies and anti-N IgG index were also non-significant after adjusting for age. Similarly, there was no significant association between anti-N IgG index and the sum of the standardized 91 AABs, adjusted for sex and age.

## Discussion

In this study, comprehensive profiling of AAB activation in 177 HCs with prior SARS-CoV-2 infection revealed several important sex-specific findings of interest. First, a surprisingly large number of the diverse autoantibodies assayed were differentially activated in males compared to females. Among previously infected individuals who were asymptomatic, the breadth of AABs response was more prominent in women than in men; by contrast, among previously infected individuals who experienced at least a mild burden of symptoms, the extent of antibody response was far more pronounced in men. Second, we found AAB responses to symptom clusters were also sex-specific, with certain associations seen more prominently in men compared to women, across the range of symptom burden. Third, we found that several autoantibodies, including ACE2, CSF2, LYZ, MDA5, MOV10, SNRPB, SOX13 and SRP19 significantly associated with higher overall symptom timing score, which may indicate that synthesis of these AABs is triggered at the beginning of the infections and correlated to symptoms that were reported closer to the blood draw. Finally, we observed these sex-specific AABs associations up to 6 months following symptomatology, indicating that SARS-CoV-2 triggers a complement of AABs responses that persists over time – in a sex-specific manner and irrespective of illness severity.

The current study expands from prior work in several ways. Extending from previous studies reporting on presence of post-COVID-19 autoimmunity [3, 23], we employed a broad array of antibodies to 91 distinct antigens previously linked to classic autoimmune conditions. Our results reveal a remarkable sex-specific prevalence and selectivity of the AABs response to SARS-CoV-2. Confirming and extending from the findings from prior reports, we found that a majority of our previously infected study participants had detectable circulating AABs against antigens such as ACE2, AQP4, C3, CHD4, CHGA, CXCL8, DBT, ECE1, ELANE, EXOSC10, HARS, HIST1H4A, IGF1R, INS, MOV10, MX1, PRTN3, RNF41, RPLP2, S100A9, SET, SNRPD1, SOX13, TG, TGFB1, TOP1, UBTF. Intriguingly, a distinct set of AABs to 59 antigens were highly correlated with reported symptoms in the male population, while another set of AABs to only 38 antigens were associated with symptoms in females. Notably, in males, we observed AABs associated with symptoms at a high frequency (≥ 6 symptoms) as well as at a moderate frequency (≥ 4 symptoms). The high frequency associated AABs included SNRPB, a ribonucleoprotein that is widely prevalent in human SLE [24]. The moderate frequency associated AABs included MOV10, CHGA, CHD4, HIST1H4A, ACE2, IFNA6, LYZ, RNF41. Importantly, both MOV10 and IFNA6 have been reported in patients infected with COVID-19 [21, 25]. In females, we observed an overall lower frequency of significant symptoms associated AABs when compared to males. The 3 most prominent symptoms in females were associated with AABs to DBT and ROS1. Interestingly, AABs to DBT have been associated with lung cancer [26]. Importantly, a number of AABs can be classified as implicated more frequently with systemic disease traits (i.e., multi-organ or multi-system) which may be particularly relevant to the more non-specific symptoms such as fatigue, fever, rashes, cold or allergy-type symptoms, weight loss, and muscular weakness.

Intriguingly, despite greater co-occurrence of distinct symptoms in females and overall between-sex similarity in the most highly frequent symptom types, males demonstrated a broader AAB response with a greater number of distinct AAB clusters identified across particularly the milder levels of symptom burden. The sex specificity of triggered AABs reactivity in association with either distinct symptoms, or symptom clusters, may be related not only to sex differences in acute illness but also in post-acute and chronic clinical syndromes experienced by a substantial number of individuals recovering from COVID-19 [27].

While apparently paradoxical at the outset, our sex-specific findings are congruent with ongoing emerging data regarding potential mechanisms underlying sex differences in the susceptibility and response to SARS-CoV-2. Early studies reported that while men and women have similar prevalence, men with COVID-19 are at greater risk for worse outcomes and death independent of age [28, 29]. Consistent with these findings, conventional inflammatory markers are founded to be more substantially elevated in men compared to women who are hospitalized for COVID-19 [30]. Accordingly, males in our study had greater AAB reactivity after adjusting for age. For classic autoimmune disease, clinical prevalence and incidence of autoimmune diseases tend to exhibit sex-specific patterns based on pathobiology. Male-predominant autoimmune diseases usually manifest clinically (i.e., show signs and symptoms of clinical disease) prior to age 50 and are characterized by acute inflammation and a Th1-type response, whereas autoimmune diseases with a greater incidence in females that occur early in life have a clearer antibody-mediated pathology. Autoimmune diseases that have a greater incidence in females and also appear clinically later in life tend to present with evidence of chronic pathology, fibrosis, and increased numbers of autoantibodies are present [31].

The conventional sex bias seen for classic autoimmune diseases has been attributed in part to women who have a generally stronger cellular and humoral immunity, higher levels of circulating antibodies, more numerous circulating CD4+ T cells, and more robust cytokine production in response to immune stressors such as infection [32, 33]. By contrast, males are now recognized as more vulnerable to the immune-modulated effects of active SARS-CoV-2 infection likely due to multiple mechanisms (e.g. lower immune cell expression of TLR7, lower observed antibody response, and lower interleukin mediated tissue resilience and tissue repair activity) [33].

Our findings revealed a broad anti-viral immune response detected in previously COVID-19 infected individuals that included reactivity to proteins with molecular homology with SARS-CoV-2 including: MOV10, a protein interaction partner of SARS-CoV-2; adenylate kinase 4 (AK4), a Y14-protein homologue of SARS-CoV-2); and, La ribonucleoprotein domain family member 1 (LARP1), a N protein interaction partner of SARS-CoV-2. Importantly, the autoantibody responses seen across all seropositive individuals were not significantly correlated with concurrent elevations in anti-N IgG index, thus indicating orthogonal immune activation. Notably, only AK4 has been described as SARS-CoV-2 homolog that correlates with anti-N IgG index. However, of the total 12 homologs or interaction partners of SARS-CoV-2 detected by the array, a significantly elevated response to the MOV10 antigen was detected in males and in correlation with the symptom of fever. By contrast, a more prominent AAB response to LARP1 was elevated in females.

These findings demonstrate that SARS-CoV-2 directly triggers the development of AABs not limited to structural proteins similar to SARS-CoV-2. Importantly, our study also demonstrates for the first time an autoimmune activation in females compared to males following asymptomatic infection. As context, the stability of AABs in classic autoimmune diseases is known to vary substantially, with some autoantibodies fluctuating with flares of disease, while others remain stable.

We can speculate that the preponderance of AABs positivity in females—in the absence of symptomatic or recognized infection—represents initiation or proliferation and then persistence of self-reactive immunity with implications for post-acute chronic immune-driven disease states. These findings may be particularly relevant to rapidly accumulating evidence of the post-acute SARS-CoV-2 syndromes (e.g. “long-haul COVID”) that can emerge even weeks to months following resolution of mild or asymptomatic infection and with clinical manifestations that appear to differ in women compared to men [27].

The existence of autoantibodies within normal healthy individuals has been already shown by other investigators [34]. The fact that across the breadth of AABs assayed in our healthy control sample, titers were also male predominant suggesting that larger population-based screening studies are needed to clarify our understanding of sex differences in basal AAB variation in the absence of clinical disease. Importantly, variations in the AAB titers found in the HCWs were different than those seen in healthy control subjects. In the latter group, the most dominant AAB was for granulocyte–macrophage colony-stimulating factor, also known as colony-stimulating factor 2 (CSF2), well-known to be a regulator of monocyte/macrophage differentiation. By contrast, AAB against CSF2 in the HCWs was barely reactive in the male population and were seen to be upregulated in female in asymptomatic group.

Several limitations of our study merit consideration. Our cohort includes HCWs from a single center who volunteered and responded to surveys, potentially limiting generalizability. Given this study’s focus on the milder spectrum of COVID-19 disease presentation and its sequelae, gradation of illness severity was determined based on self-reported symptom burden and very few of our cases (1.7%) required hospitalization and none were treated with immune-modulating therapies for their COVID-19 illness. Thus, further studies are needed to evaluate generalizability of our findings to populations that experienced more severe COVID-19 illness requiring targeted treatments. We were unable to verify exact dates of prior COVID-19 infection due to lack of universal PCR testing available during the initial phases of the pandemic and, thus, associations between timing of infection, symptoms, and AAB response could not be precisely analyzed. We have a relatively small number of male subjects (n = 63) that may have limited the ability to detect potential additional predicators of post-COVID autoimmunity; thus, further investigations of larger sized samples are needed. Our sampling included a 1:3 ratio of controls to cases, and a further balanced ratio of controls to cases in future larger sized studies could offer even greater statistical power for detecting additional potential associations of interest and importance. Although this was a prospective study, the survey method involved requesting participants to self-report symptoms occurring up to 6 months prior to the blood draw, contributing to potential recall bias. Whether examined subjectively or objectively, symptomatology can vary not only between but also within individuals over time. Similarly, the status of AAB reactivity may change over time and in relation to the timing of initial or repeated exposures. Thus, future longitudinal studies are warranted to understand temporal trends in similarly measured exposure and outcomes.

In summary, this comprehensive study of AABs to a wide array of antigens found that male sex carries the risk of diverse autoimmune activation following symptomatic COVID-19 illness, whereas female sex carries risk for a distinct profile of autoimmune activation following asymptomatic SARS-CoV-2 exposure. Importantly, both sets of sex-specific AAB reactivity patterns were found to persist up to 6 months following associated symptomatology. Further understanding of the nature of triggered and persistent AAB activation among individuals who are exposed to SARS-CoV-2—and vulnerable to its potentially morbid clinical sequelae—will be essential for developing effective interventions and therapeutics.

## Supplementary Information

**Additional file 1.** Supplementary Materials.

## Data Availability

Requests for de-identified data may be directed to the corresponding authors (JEVE, SC, JFB) and will be reviewed by the Office of Research Administration at Cedars-Sinai Medical Center prior to issuance of data sharing agreements. Data limitations are designed to ensure patient and participant confidentiality.
