# Supplementary material for: Paradoxical sex-specific patterns of autoantibody response to SARS-CoV-2 infection
Source: J Transl Med. 2021 Dec 30;19:524. doi: 10.1186/s12967-021-03184-8 (PMC8716184; doi:10.1186/s12967-021-03184-8)

SUPPLEMENTAL MATERIAL

Paradoxical Sex-Specific Patterns of Autoantibody Response to SARS-CoV-2 Infection

Table S1. Composition of the AABs array.

No.	Protein Ref ID	Gene Symbol	Gene Name	Associated Traits
1	0105507583	NCL	Nucleolin (Protein C23)	R1ab protein homologue
2	0104741802	SRSF1	Serine/arginine-rich splicing factor 1	N-protein homologue
3	0104741211	RAE1	Ribonucleic acid export 1	ORF6 interaction partner
4	0104743520	EIF4H	Eukaryotic translation initiation factor 4H	NSP9 interaction partner
5	0105483772	MOV10	Mov10 RISC complex RNA helicase	N protein interaction partner
6	1043144426	NUMA1	Nuclear mitotic apparatus protein 1	pSS, CTD
7	0105510253	KDM6B	Lysine demethylase 6B	SLE, SSc
8	0105482537	ANXA2	Annexin A2	
9	0105488388	TLE1	Transducin-like enhancer of split 1	NSP13 interaction partner
10	1066861231	SOX13	Transcription factor SOX-13	PBC, type 1 diabetes
11	1066861238	SLC30A8	Solute carrier family 30 member 8	Type 1 diabetes
12	1066860558	SERPINB4	Serpin family B member 4	Lung-specific protein
13	1066858160	SERPINB3	Serpin family B member 3	Lung-specific protein
14	1066858257	IL4	Interleukin 4	Lung fibrosis
15	1066558274	SRP19	Signal recognition particle 19kDa	NSP8 interaction partner
16	0104741509	SET	Protein SET (HLA-DR-associated protein II)	R1a-Protein homologue
17	0105481639	GRP	Gastrin releasing peptide	Lung fibrosis
18	1105172472	AK4	Adenylate kinase 4, mitochondrial	Y14-Protein homologue
19	1066866713	HNRNPA2B1	Heterogeneous nuclear ribonucleoprotein A2/B1	RA, SLE
20	0104740269	LARP1	La ribonucleoprotein domain family, member 1	N protein interaction partner
21	1043139412	S100A9	S100 calcium binding protein A9	
22	0104745439	MIF	Macrophage migration inhibitory factor	
23	1047888619	UBTF	Upstream binding transcription factor, RNA polymerase I	CTD, SSc, SLE, SjS
24	1066838756	LTF	Lactotransferrin	Vasculitis
25	1066858459	S100A8	S100 calcium binding protein A8	
26	1066527806	PPL	Periplakin	Antigen for autoimmunity in idiopathic pulmonary fibrosis
27	1066559705	IL17A	Interleukin 17A	SLE, lung fibrosis
28	1066858740	IFNA4	Interferon alpha 4	SLE
29	0105489152	RNF41	E3 ubiquitin-protein ligase NRDP1 (RING finger protein 41)	NSP15 interaction partner
30	0105489248	VEGFA	Vascular endothelial growth factor A	

31	1066561916	HIST1H4A	Histone cluster 1 H4 family member a	Drug-induced lupus (DIL)
32	1066838464	ECE1	Endothelin converting enzyme 1	Biomarker pulmonary fibrosis
33	1066859699	ICA1	Islet cell autoantigen 1	Type 1 diabetes
34	0104744361	CHGA	Chromogranin A	
35	0105483201	NPM1	Nucleophosmin	SLE, SSc with pulmonary arterial hypertension
36	1066562106	CSF2	Colony stimulating factor 2	SLE, lung fibrosis
37	1066560001	CXCL8	C-X-C motif chemokine ligand 8	
38	1066859503	ELANE	Elastase, neutrophil expressed	Vasculitis
39	1043136644	IL6	Interleukin 6	SLE
40	1043140678	MX1	Interferon-induced GTP-binding protein Mx1	SLE, Chronic Pulmonary Diseases
41	1066562002	IL10	Interleukin 10	SLE, lung fibrosis
42	1066859715	CTSG	Cathepsin G	Vasculitis
43	1043140754	CHD3	Chromodomain helicase DNA binding protein 3	Myositis
44	1043144332	AQP4	Aquaporin 4	NMO
45	1066859023	LYZ	Lysozyme	Vasculitis
46	1066858547	IFNA6	Interferon alpha 6	SLE
47	1166361472	IGF1R	Insulin like growth factor 1 receptor	Autoimmune thyroiditis
48	1197785315	VIM	Vimentin	SLE, RA, CTD
49	1197682501	ROS1	ROS proto-oncogene 1, receptor tyrosine kinase	Lung-specific protein
50	1208494664	VEGFC	Vascular endothelial growth factor C	
51	1208494784	ENO1	Enolase 1	Vasculitis
52	0184571702	SRP54	Signal recognition particle 54	Myositis
53	0184571651	DBT	Dihydrolipoamide branched chain transacylase E2	Primary Biliary Cirrhosis (PBC)
54	0184571673	Chd4	Chromodomain helicase DNA binding protein 4	Myositis
55	0184649040	DLAT	Dihydrolipoamide S-acetyltransferase	Primary Biliary Cirrhosis (PBC)
56	0184571698	MDA5	Melanoma differentiation-associated protein 5	
57	0184571630	TRIM33	Tripartite motif containing 33	Myositis
58	0184571670	EXOSC10	Exosome component 10	Myositis/SSc
59	0184571689	SNRPB	Small nuclear ribonucleoprotein polypeptides B and B1	SLE
60	0184571639	SNRPC	Small nuclear ribonucleoprotein polypeptide C	SLE, CTD
61	0184571711	SNRNP70	Small nuclear ribonucleoprotein U1 subunit 70	SLE, CTD
62	0184571645	SSB	Sjogren syndrome antigen B	SSB / pSS, SLE
63	0184571685	SmD3	Small nuclear ribonucleoprotein D3	

64	0184571664	SNRPA	Small nuclear ribonucleoprotein polypeptide A	SLE, CTD
65	1058284508	TOP1	Topoisomerase (DNA) I	Diffuse SSc
66	0184571620	TROVE2	TROVE domain family member 2	SSA / SLE, SSB, CTD
67	0184571617	SNRPD1	Small nuclear ribonucleoprotein D1 polypeptide	SLE
68	0209387966	IFNA2	Interferon alpha-2	SLE/RA
69	0184648993	MPO	Myeloperoxidase	Vasculitis
70	0184649015	Sp100	SP100 nuclear antigen	Primary Biliary Cirrhosis (PBC)
71	0184649061	PRTN3	Proteinase 3	Vasculitis
72	1058284066	CENPB	Centromere protein B	Limited SSc
73	0201790353	TGFB1	Transforming growth factor beta 1	
74	0221992867	IFNW1	Interferon omega 1	SLE
75	0236191321	TPO	Thyroid peroxidase	Autoimmune thyroiditis
76	0348725826	PTPRN	Protein tyrosine phosphatase receptor type N	Type 1 diabetes
77	0348725809	TSHR	Thyroid stimulating hormone receptor	Autoimmune thyroiditis
78	1086925499	ACE2	Angiotensin I converting enzyme 2	
79	0184571695	HARS	Histidyl-tRNA synthetase	Anti-synthetase syndrome
80	0184649012	APOH	Apolipoprotein H	Anti-phospholipid syndrome (APS, SLE)
81	0184570981	RPLP2	Ribosomal protein lateral stalk subunit P2	SLE
82	0184571682	TRIM21	Tripartite motif containing 21	SSA / SLE, pSS, CTD
83	0201790340	SPP1	Secreted phosphoprotein 1	
84	1049727854	C3	Complement C1q B chain	SLE
85	0348725837	GAD65	Glutamate decarboxylase 2	
86	0348725819	TG	Thyroglobulin	Autoimmune thyroiditis
87	0365576846	INS	Insulin	Type 1 diabetes
88	0174988610	CTLA4	Cytotoxic T-lymphocyte associated protein 4	
89	0184649087	DLST	Dhydrolipoamide S-succinyltransferase	Primary Biliary Cirrhosis (PBC)
90	0105482432	NCOA6	Nuclear receptor coactivator 6	
91	0349974412	CADM3	Cell adhesion molecule 3	Membranous lupus nephritis
91	Control_BSA		Control_BSA	
92	Control_Ecoli		Control_Ecoli	
93	Control_huIgGhi		Control_huIgGhi	
94	Control_huIgGlo		Control_huIgGlo	
95	Control_huIgM		Control_huIgM	
96	Control_huIgA		Control_huIgA	

Table S2. Demographic and clinical characteristics of 177 HCWs

	**Overall (N=177)**	**Women (N=115)**	**Men (N=62)**	**P value**
**Age (median [IQR])**	35.00 [30.00, 44.00]	35.00 [30.00, 46.50]	34.50 [29.25, 40.75]	0.529
**Race/Ethnicity (%)**				
Hispanic/Latinx	50 (28.2)	34 (29.6)	16 (25.8)	0.531
Non-Hispanic Asian	46 (26.0)	30 (26.1)	16 (25.8)	
Non-Hispanic Black	15 (8.5)	11 (9.6)	4 (6.5)	
Non-Hispanic White	56 (31.6)	32 (27.8)	24 (38.7)	
Other	10 (5.6)	8 (7.0)	2 (3.2)	
**Medical Conditions**				
Cancer	2 (1.2)	2 (1.8)	0 (0.0)	0.797
Cardiovascular	2 (1.2)	1 (0.9)	1 (1.7)	1.000
Chronic Obstructive Pulmonary Disease	0 (0)	0 (0)	0 (0)	NA
Diabetes Mellitus	5 (2.9)	3 (2.7)	2 (3.3)	1.000
Hypertension	19 (11.0)	13 (11.4)	6 (10.2)	1.000
Immune	3 (1.7)	3 (2.7)	0 (0.0)	0.504
Prior diagnosis of COVID-19	68 (38.4)	42 (36.5)	26 (41.9)	0.586
Smoking	2 (1.1)	1 (0.9)	1 (1.6)	1.000
Vaping	14 (7.9)	6 (5.2)	8 (12.9)	0.130
**Reported symptoms**				
Chest pain	36 (20.3)	24 (20.9)	12 (19.4)	0.966
Chills	86 (48.6)	52 (45.2)	34 (54.8)	0.287
Conjunctivitis	11 (6.2)	5 (4.3)	6 (9.7)	0.282
Dry cough	100 (56.5)	64 (55.7)	36 (58.1)	0.881
Productive cough	44 (24.9)	32 (27.8)	12 (19.4)	0.288
Diarrhea	53 (29.9)	31 (27.0)	22 (35.5)	0.313
Fatigue	117 (66.1)	76 (66.1)	41 (66.1)	1.000
Fever	78 (44.1)	47 (40.9)	31 (50.0)	0.313
Headache	110 (62.1)	73 (63.5)	37 (59.7)	0.738
Loss of appetite	68 (38.4)	49 (42.6)	19 (30.6)	0.162
Muscle aches	101 (57.1)	66 (57.4)	35 (56.5)	1.000
Nasal congestion	93 (52.5)	60 (52.2)	33 (53.2)	1.000
Nausea	47 (26.6)	37 (32.2)	10 (16.1)	0.033
Runny nose	76 (42.9)	55 (47.8)	21 (33.9)	0.103
Shortness of breath	57 (32.2)	33 (28.7)	24 (38.7)	0.233
Skin changes	13 (7.3)	9 (7.8)	4 (6.5)	0.974
Smell and/or taste	92 (52.0)	61 (53.0)	31 (50.0)	0.819
Sneezing	83 (46.9)	56 (48.7)	27 (43.5)	0.619
Sore throat	71 (40.1)	48 (41.7)	23 (37.1)	0.660
Vomiting	16 (9.0)	9 (7.8)	7 (11.3)	0.623
**Symptom severity**				0.982
Asymptomatic	23 (13.0)	15 (13%)	8 (12.9%)	
Mild symptom burden	64 (36.2)	41 (35.7%)	23 (37.1%)	
More than mild symptom burden	90 (50.8)	59 (51.3%)	31 (50%)	

Table S3. Sex-specific clustering of autoantibody responses, by sex and symptom burden

Men, Asymptomatic

Cluster	AAB
1	MOV10 IL4 SET S100A9 IFNA4 NPM1 CSF2 LYZ IFNA6 IGF1R ROS1 DLAT SNRNP70 TROVE2 IFNA2 PRTN3 IFNW1 ACE2 HARS RPLP2 SPP1 C3 TG CTLA4 DLST NCOA6
2	SOX13 MPO TPO TSHR APOH
3	KDM6B LARP1 PPL AQP4 VEGFC SRP54 DBT SNRPB SNRPC SNRPA SNRPD1 GAD65
4	SRSF1 RAE1 NUMA1 SERPINB4 GRP HNRNPA2B1 S100A8 ICA1 CHGA IL6 MX1 CHD3 MDA5 TRIM33 SSB TGFB1 TRIM21 INS
5	NCL EIF4H ANXA2 TLE1 SLC30A8 SERPINB3 SRP19 AK4 MIF UBTF LTF IL17A RNF41 VEGFA HIST1H4A ECE1 CXCL8 ELANE IL10 CTSG VIM ENO1 CHD4 EXOSC10 SMD3 TOP1 SP100 CENPB PTPRN CADM3

Women, Asymptomatic

Cluster	AAB
1	IL4 S100A9 IFNA4 NPM1 IL10 LYZ IGF1R DLAT SNRNP70 TROVE2 PRTN3 IFNW1 HARS RPLP2 SPP1 C3 TG CTLA4 DLST
2	SOX13 GRP HNRNPA2B1 LARP1 S100A8 PPL IL6 CHD3 SRP54 DBT SNRPB SNRPC SSB SNRPA SNRPD1 TPO TSHR GAD65
3	MOV10 ANXA2 TLE1 SET LTF IL17A RNF41 VEGFA ECE1 CSF2 CTSG IFNA6 VIM ROS1 TRIM33 IFNA2 MPO SP100 CENPB PTPRN ACE2 APOH NCOA6 CADM3
4	NCL SRSF1 RAE1 EIF4H NUMA1 KDM6B SLC30A8 SERPINB4 SERPINB3 SRP19 AK4 MIF UBTF HIST1H4A ICA1 CHGA CXCL8 ELANE MX1 AQP4 VEGFC ENO1 CHD4 MDA5 EXOSC10 SMD3 TOP1 TGFB1 TRIM21 INS

Men, After Mild Symptoms

Cluster	AAB
1	TPO
2	SRSF1 KDM6B GRP HNRNPA2B1 LARP1 S100A8 MDA5 SNRPD1
3	VIM ROS1 VEGFC ENO1
4	MPO APOH NCOA6
5	NCL RAE1 EIF4H SERPINB4 SERPINB3 SRP19 AK4 MIF UBTF ICA1 CXCL8 ELANE MX1 CHD4 TRIM33 EXOSC10 SMD3 TOP1 SP100 TGFB1 INS
6	MOV10 NUMA1 SLC30A8 LTF CHGA PTPRN CADM3
7	IL4 S100A9 IFNA4 IL10 LYZ IGF1R DLAT SNRNP70 TROVE2 PRTN3 IFNW1 HARS RPLP2 SPP1 C3 TG DLST
8	PPL IL6 DBT SNRPB SNRPC TSHR GAD65
9	SOX13 CHD3 AQP4 SRP54 SSB SNRPA TRIM21
10	ANXA2 TLE1 SET IL17A RNF41 VEGFA HIST1H4A ECE1 NPM1 CSF2 CTSG IFNA6 IFNA2 CENPB ACE2 CTLA4

Women, After Mild Symptoms

Cluster	AAB
1	KDM6B SOX13 GRP LARP1 PPL CHD3 SRP54 DBT SNRPB SNRPC SSB SNRPA SNRPD1 TPO TSHR GAD65
2	MOV10 ANXA2 IL4 SET S100A9 LTF IL17A IFNA4 RNF41 ECE1 NPM1 CSF2 IL10 CTSG LYZ IFNA6 IGF1R VIM ROS1 DLAT SNRNP70 TROVE2 IFNA2 PRTN3 CENPB IFNW1 PTPRN ACE2 HARS RPLP2 SPP1 C3 TG CTLA4 DLST NCOA6 CADM3
3	NCL SRSF1 RAE1 EIF4H NUMA1 TLE1 SLC30A8 SERPINB4 SERPINB3 SRP19 AK4 HNRNPA2B1 MIF UBTF S100A8 VEGFA HIST1H4A ICA1 CHGA CXCL8 ELANE IL6 MX1 AQP4 VEGFC ENO1 CHD4 MDA5 TRIM33 EXOSC10 SMD3 TOP1 MPO SP100 TGFB1 APOH TRIM21 INS

Men, After More than Mild Symptoms

Cluster	AAB
1	MOV10 CHGA
2	SET NPM1 DLAT SNRNP70 IFNA2 PTPRN HARS RPLP2 CTLA4 NCOA6
3	SRSF1 RAE1 KDM6B SERPINB4 SERPINB3 HNRNPA2B1 ICA1 IL6 TRIM21
4	NCL AK4 MIF UBTF CXCL8 ELANE MX1 CHD4 EXOSC10 SMD3 TOP1 INS
5	EIF4H RNF41 VEGFA HIST1H4A ECE1 VIM ROS1 ENO1 TRIM33 SP100 CENPB TGFB1
6	S100A9 IFNA4 LYZ IGF1R TROVE2 PRTN3 IFNW1 SPP1 C3 TG DLST
7	GRP S100A8 CHD3 AQP4 MDA5
8	TPO TSHR
9	ANXA2 TLE1 LTF IL17A
10	NUMA1 SLC30A8 SRP19 LARP1
11	SNRPB SNRPC SNRPA SNRPD1
12	IL4 CSF2 IL10 CTSG IFNA6 ACE2 CADM3
13	SOX13 PPL SRP54 DBT SSB GAD65
14	MPO APOH
15	VEGFC

Women, After More than Mild Symptoms

Cluster	AAB
1	MOV10 IL4 CSF2 CTSG IFNA6 ACE2
2	SRSF1 KDM6B SERPINB4 GRP SNRPC
3	ANXA2 TLE1 LTF IL17A RNF41 VEGFA TRIM33 SP100 CENPB PTPRN
4	SET S100A9 IFNA4 NPM1 IL10 LYZ IGF1R IFNW1 HARS RPLP2 CTLA4
5	VIM ROS1 VEGFC ENO1
6	EIF4H NUMA1 INS
7	SLC30A8 CHGA AQP4
8	SRP54 DBT SSB SNRPA GAD65
9	LARP1 PPL SNRPB SNRPD1
10	MPO APOH
11	TSHR
12	NCL RAE1 SERPINB3 SRP19 AK4 MIF UBTF HIST1H4A CXCL8 ELANE MX1 CHD4 EXOSC10 SMD3 TOP1 TGFB1
13	SOX13
14	HNRNPA2B1 MDA5
15	TPO
16	NCOA6
17	CHD3
18	ECE1 DLAT SNRNP70 IFNA2 CADM3
19	TROVE2 PRTN3 SPP1 C3 TG DLST
20	S100A8
21	ICA1 IL6 TRIM21

Table S4. Associations of autoantibody reactivity with symptoms in men. Beta coefficients from age-adjusted regression analysis comparing males with a specific symptom burden to males without the same symptom are shown. The last 3 columns display results from age-adjusted analyses comparing males with different levels of symptoms burden to the pre-pandemic healthy control group.

Beta	Chest pain	Chills	Conjunctivitis	Cough productive	Diarrhea	Fatigue	Fever	Headache	Loss of appetite	Muscle aches	Nasal congestion	Nausea	Runny nose	Shortness of breath	Skin	Smell taste	Sneezing	Sore throat	Asymptomatic	Mild	More than mild
**ACE2**	0.467	0.202	0.164	0.602	0.461	0.232	0.091	0.161	0.258	0.486	0.738	0.015	0.634	0.536	-0.261	0.541	0.445	0.338	-0.428	0.362	0.470
**AK4**	0.117	0.133	-0.527	0.634	0.293	0.337	-0.016	0.187	0.224	0.201	0.411	0.132	0.207	0.183	-0.109	0.198	0.206	0.235	-0.372	-0.008	0.128
**ANXA2**	-0.168	-0.280	-0.101	0.604	0.250	0.126	-0.084	-0.114	0.390	-0.098	0.461	0.180	0.259	0.298	-0.256	0.403	0.325	0.203	0.138	0.385	0.431
**APOH**	0.483	0.095	0.528	0.579	0.153	-0.079	-0.014	-0.063	0.220	0.186	0.059	0.693	0.147	0.156	-0.002	-0.525	0.257	0.549	0.716	0.287	0.509
**AQP4**	-0.483	-0.382	-0.121	-0.448	-0.128	-0.425	-0.281	-0.419	-0.339	-0.189	-0.125	-0.725	-0.113	-0.626	-0.589	-0.468	-0.065	-0.405	0.509	0.590	-0.078
**C3**	0.094	0.119	0.503	0.505	0.420	0.386	-0.107	-0.021	0.427	0.125	0.490	-0.444	0.361	0.367	-0.218	0.640	0.292	-0.002	-0.195	0.509	0.472
**CADM3**	-0.090	-0.168	-0.207	0.375	-0.300	-0.156	-0.046	-0.737	-0.144	0.160	0.334	-0.236	0.507	0.084	-0.276	0.090	0.179	0.008	0.596	0.709	0.622
**CHD4**	0.137	0.032	-0.221	0.882	0.228	0.147	-0.090	-0.127	0.370	0.003	0.627	0.009	0.452	0.109	-0.414	0.285	0.573	0.508	-0.228	0.406	0.415
**CHGA**	0.605	0.364	0.227	0.642	0.327	0.094	0.135	0.045	0.256	0.170	0.042	0.446	-0.132	0.584	0.453	0.163	0.276	0.578	-0.295	-0.422	0.085
**CSF2**	0.476	0.136	-0.155	0.654	0.591	0.176	0.047	0.245	0.198	0.471	0.518	0.342	0.430	0.365	-0.374	0.391	0.317	0.324	-0.652	0.212	0.338
**CTLA4**	-0.275	-0.174	0.257	0.428	0.198	0.215	-0.153	-0.231	0.195	0.017	0.682	-0.370	0.563	0.448	-0.244	0.710	0.420	-0.083	-0.148	0.688	0.473
**CTSG**	0.214	-0.043	-0.307	0.694	0.326	0.101	-0.056	0.085	0.159	0.289	0.723	0.102	0.684	0.322	-0.114	0.432	0.457	0.236	-0.248	0.307	0.496
**CXCL8**	0.287	0.100	-0.682	0.866	0.208	0.118	-0.094	0.110	0.254	0.377	0.589	0.204	0.522	0.311	-0.370	0.313	0.424	0.605	-0.429	-0.030	0.268
**DBT**	0.047	-0.285	-0.215	0.144	-0.245	-0.289	-0.157	-0.326	0.003	-0.391	0.262	0.253	0.570	-0.280	0.781	-0.314	0.125	-0.279	0.293	0.386	-0.100
**DLAT**	0.063	-0.107	0.174	0.190	0.290	0.094	-0.206	-0.114	0.002	0.108	0.262	-0.291	0.219	0.001	0.435	0.439	0.219	-0.054	0.295	0.286	0.405
**ECE1**	0.424	0.096	0.107	1.132	0.327	0.397	0.153	0.065	0.513	0.059	0.631	0.100	0.507	0.457	0.678	0.223	0.579	0.247	-0.610	-0.092	-0.078
**EIF4H**	0.069	0.067	-0.150	0.612	0.187	0.099	0.077	0.117	0.307	-0.156	0.421	-0.032	0.384	0.383	0.087	0.174	0.424	0.006	-0.142	0.492	0.434
**ELANE**	-0.177	-0.190	-0.473	0.319	0.115	0.258	-0.002	0.117	-0.162	0.273	0.606	-0.196	0.558	0.233	0.525	0.596	0.381	0.039	-0.061	0.325	0.645
**EXOSC10**	0.059	0.261	0.506	0.785	-0.111	0.176	-0.115	0.027	0.074	0.166	0.153	0.041	0.156	0.147	-0.349	0.367	0.244	0.324	-0.242	0.468	0.301
**GRP**	0.438	0.342	0.030	1.030	0.347	0.216	0.211	0.380	0.360	0.208	0.228	0.702	0.222	0.203	0.285	0.093	0.329	0.483	-0.532	0.156	-0.012
**HARS**	-0.059	0.149	0.350	0.620	0.273	0.393	-0.071	-0.074	0.260	0.155	0.500	-0.363	0.305	0.287	-0.247	0.497	0.326	0.178	-0.035	0.572	0.563
**HIST1H4A**	0.098	0.226	-0.329	0.874	0.167	0.288	-0.133	0.021	0.098	0.297	0.550	0.176	0.580	0.291	-0.379	0.312	0.316	0.479	-0.256	0.111	0.220
**ICA1**	0.006	-0.035	-0.713	0.863	0.182	0.297	0.018	0.229	0.130	0.200	0.387	0.363	0.017	0.406	-0.050	0.288	0.340	0.365	-0.161	0.037	0.455
**IFNA4**	-0.064	-0.035	0.465	0.476	0.282	0.381	0.007	-0.036	0.324	0.156	0.649	-0.321	0.597	0.285	0.316	0.624	0.464	-0.033	-0.300	0.533	0.481
**IFNA6**	0.274	0.144	0.375	0.650	0.211	0.336	-0.124	0.050	0.215	0.360	0.651	-0.002	0.723	0.328	-0.077	0.535	0.429	0.268	-0.633	0.120	0.178
**IGF1R**	-0.051	-0.094	0.285	0.294	0.449	0.212	-0.016	-0.029	0.206	0.225	0.517	-0.423	0.340	0.255	-0.292	0.649	0.188	-0.121	-0.269	0.449	0.403
**IL17A**	0.207	-0.128	-0.369	0.761	0.490	0.078	0.087	0.039	0.539	0.039	0.449	0.709	0.046	0.453	-0.249	0.077	0.378	0.262	0.043	-0.215	0.118
**IL4**	0.244	-0.077	-0.101	0.557	0.528	-0.075	-0.012	-0.317	0.460	0.261	0.659	0.048	0.532	0.294	-0.337	0.202	0.686	0.082	-0.233	0.263	0.380
**IL6**	0.251	-0.105	-0.038	0.573	-0.103	-0.200	0.079	-0.622	0.052	-0.338	0.455	0.318	0.256	0.223	-0.259	0.121	0.401	0.134	0.242	0.753	0.316
**INS**	-0.377	-0.319	-0.303	0.855	-0.308	-0.068	-0.292	-0.361	0.111	-0.210	0.246	-0.089	0.310	0.023	-0.165	-0.025	0.292	-0.126	0.582	0.800	0.434
**LYZ**	0.126	0.055	0.420	0.634	0.348	0.270	-0.017	0.079	0.229	0.345	0.823	-0.076	0.773	0.419	-0.020	0.554	0.573	0.173	-0.429	0.326	0.478
**MDA5**	0.233	0.005	-0.123	1.281	0.256	0.084	-0.190	0.070	0.298	0.020	0.578	0.479	0.437	0.232	0.431	0.319	0.412	0.676	-0.138	0.534	0.550
**MIF**	0.012	0.193	-0.210	0.957	0.103	0.256	0.008	-0.121	0.192	0.233	0.473	0.421	0.499	0.249	-0.066	0.252	0.476	0.690	0.271	0.682	0.826
**MOV10**	0.197	0.058	0.950	0.737	0.738	0.331	0.268	0.033	0.671	0.388	0.734	0.583	0.428	0.390	0.717	0.298	0.552	0.620	-0.338	0.497	0.678
**MX1**	0.096	0.003	-0.567	1.019	-0.109	-0.089	-0.036	-0.484	-0.010	0.005	0.615	-0.044	0.569	0.296	-0.452	0.406	0.449	0.214	0.299	0.648	0.678
**NCL**	0.057	0.204	-0.564	0.884	0.273	0.354	0.199	0.049	0.433	0.248	0.569	0.375	0.218	0.477	-0.445	0.278	0.277	0.561	-0.105	0.407	0.700
**NPM1**	-0.148	-0.256	0.074	0.750	0.099	0.265	-0.205	-0.238	0.174	0.129	0.407	-0.250	0.449	0.152	0.195	0.554	0.354	0.025	0.029	0.648	0.495
**PRTN3**	-0.084	-0.125	0.152	0.357	0.022	0.062	-0.054	-0.345	-0.045	0.167	0.493	0.092	0.432	0.222	-0.044	0.104	0.499	0.081	-0.198	0.320	0.263
**RAE1**	-0.226	-0.569	-0.854	0.209	0.352	-0.317	-0.250	-0.180	0.355	-0.187	0.403	0.354	0.446	-0.065	0.222	-0.167	0.363	-0.101	0.570	0.389	0.370
**RNF41**	0.017	0.063	-0.141	0.638	0.118	0.002	-0.011	-0.210	0.184	0.033	0.533	-0.079	0.545	0.304	-0.451	0.480	0.422	0.106	0.236	0.605	0.636
**RPLP2**	0.020	0.045	0.324	0.492	0.274	0.231	-0.037	-0.175	0.353	0.187	0.554	-0.052	0.523	0.413	-0.072	0.528	0.460	0.071	0.045	0.310	0.475
**S100A9**	-0.254	0.066	0.844	0.398	0.315	0.163	-0.027	-0.228	0.343	-0.005	0.560	-0.240	0.594	0.284	-0.308	0.581	0.474	-0.107	-0.064	0.607	0.491
**SET**	0.039	-0.140	0.480	0.380	0.053	0.186	-0.359	-0.327	0.169	-0.196	0.319	-0.309	0.436	0.153	-0.324	0.574	0.315	0.194	0.000	0.661	0.353
**SLC30A8**	-0.415	-0.038	0.434	-0.175	-0.016	0.400	0.140	0.112	0.113	0.340	0.661	-0.360	0.597	0.416	0.230	0.341	0.609	0.014	-0.073	0.369	0.434
**SNRNP70**	0.429	0.108	-0.260	1.066	0.556	0.069	-0.143	-0.053	0.424	0.222	0.414	0.005	0.502	0.511	-0.436	0.568	0.363	0.260	-0.189	0.140	0.487
**SNRPA**	0.564	0.025	-0.225	0.786	0.112	-0.397	0.039	-0.120	0.214	0.069	0.169	0.234	0.408	-0.181	0.040	-0.098	0.145	0.118	0.239	-0.188	-0.284
**SNRPB**	0.834	0.549	-0.389	1.357	0.171	0.325	-0.052	0.379	0.546	0.466	0.403	0.654	0.537	0.461	-0.725	0.538	0.424	0.733	-0.344	0.039	0.544
**SNRPD1**	-0.203	-0.019	-0.332	0.212	-0.270	-0.021	-0.525	0.085	-0.054	-0.333	0.168	0.051	0.415	0.061	-0.225	0.119	0.323	0.532	0.558	0.410	0.747
**SOX13**	0.255	0.108	-0.321	0.154	0.509	0.065	0.106	-0.056	0.182	-0.126	0.711	0.244	0.441	0.174	0.341	0.148	0.790	0.265	-0.057	0.172	0.698
**SRP19**	0.248	0.237	0.742	0.787	0.344	0.516	0.003	0.285	0.429	0.445	0.562	0.243	0.512	0.357	0.165	0.302	0.362	0.329	-0.145	0.281	0.593
**SSB**	0.247	0.007	-0.054	0.511	-0.151	-0.047	-0.374	0.010	0.013	0.098	0.043	0.117	0.125	-0.038	-0.275	0.097	0.312	0.583	0.073	0.474	0.601
**TG**	-0.112	0.141	0.527	0.353	0.343	0.391	0.005	-0.121	0.364	0.078	0.378	-0.496	0.242	0.249	-0.203	0.567	0.155	-0.113	-0.163	0.685	0.481
**TGFB1**	0.288	-0.365	-0.157	0.499	-0.087	-0.231	-0.403	-0.280	0.135	-0.222	0.041	-0.060	-0.102	0.247	-1.042	-0.160	0.189	0.092	0.812	0.867	0.520
**TOP1**	-0.008	0.382	-0.635	0.816	0.008	0.436	-0.013	0.024	0.125	0.407	0.289	0.151	0.178	0.197	-0.441	0.256	0.176	0.808	0.081	0.551	0.865
**TRIM21**	-0.049	0.611	0.395	0.158	0.080	0.507	0.169	0.215	0.102	0.603	0.365	0.242	0.462	-0.107	-0.692	0.225	0.210	0.283	-0.265	0.327	0.496
**TROVE2**	-0.118	0.179	0.734	0.446	0.276	0.404	-0.231	-0.015	0.249	0.152	0.357	-0.491	0.221	0.279	-0.381	0.631	0.128	-0.077	0.041	0.613	0.492
**UBTF**	0.051	0.143	-0.408	0.775	0.025	0.195	0.040	0.013	0.088	0.164	0.407	0.099	0.296	0.240	-0.124	0.303	0.355	0.409	0.011	0.464	0.769
**VEGFA**	-0.236	-0.063	-0.681	0.665	-0.091	0.162	-0.033	-0.232	0.197	0.157	0.554	-0.014	0.657	0.113	0.123	-0.035	0.365	0.259	0.519	0.754	0.824
**VIM**	-0.191	-0.380	-0.126	-0.090	0.337	0.123	-0.010	-0.102	-0.124	0.070	0.490	-0.234	0.150	0.029	-0.298	0.448	0.065	-0.229	-0.264	0.212	-0.022

Table S5. Associations of autoantibody reactivity with symptoms in men. P values from age-adjusted regression analysis comparing males with a specific symptom burden to males without the same symptom are shown. The last 3 columns display results from age-adjusted analyses comparing males with different levels of symptoms burden to the pre-pandemic healthy control group.

P value	Chest pain	Chills	Conjunctivitis	Cough productive	Diarrhea	Fatigue	Fever	Headache	Loss of appetite	Muscle aches	Nasal congestion	Nausea	Runny nose	Shortness of breath	Skin	Smell taste	Sneezing	Sore throat	Asymptomatic	Mild	More than mild
**ACE2**	0.154	0.462	0.719	0.068	0.088	0.399	0.737	0.559	0.360	0.061	0.004	0.966	0.023	0.043	0.623	0.036	0.096	0.208	0.222	0.140	0.137
**AK4**	0.677	0.573	0.173	0.024	0.208	0.148	0.946	0.426	0.352	0.370	0.064	0.664	0.395	0.424	0.811	0.377	0.371	0.307	0.431	0.980	0.699
**ANXA2**	0.574	0.260	0.808	0.043	0.313	0.613	0.730	0.648	0.125	0.681	0.050	0.577	0.316	0.220	0.594	0.086	0.182	0.405	0.731	0.122	0.129
**APOH**	0.151	0.738	0.256	0.089	0.586	0.779	0.960	0.823	0.448	0.492	0.827	0.055	0.616	0.573	0.998	0.048	0.352	0.044	0.036	0.153	0.068
**AQP4**	0.108	0.129	0.774	0.143	0.610	0.090	0.257	0.095	0.189	0.434	0.606	0.024	0.667	0.009	0.225	0.049	0.795	0.099	0.246	0.032	0.784
**C3**	0.794	0.692	0.310	0.164	0.157	0.197	0.717	0.945	0.163	0.663	0.085	0.251	0.244	0.209	0.707	0.022	0.321	0.994	0.503	0.032	0.150
**CADM3**	0.805	0.579	0.679	0.307	0.317	0.606	0.878	0.013	0.644	0.581	0.246	0.547	0.103	0.778	0.636	0.755	0.546	0.980	0.109	0.021	0.021
**CHD4**	0.656	0.902	0.602	0.003	0.369	0.566	0.720	0.619	0.156	0.990	0.008	0.978	0.085	0.664	0.400	0.241	0.020	0.039	0.494	0.124	0.085
**CHGA**	0.041	0.144	0.585	0.032	0.186	0.708	0.584	0.859	0.319	0.478	0.861	0.167	0.611	0.015	0.347	0.495	0.260	0.016	0.498	0.095	0.792
**CSF2**	0.120	0.598	0.716	0.034	0.018	0.495	0.852	0.340	0.453	0.053	0.033	0.304	0.104	0.143	0.451	0.108	0.208	0.197	0.182	0.500	0.314
**CTLA4**	0.453	0.571	0.613	0.250	0.517	0.483	0.612	0.451	0.536	0.955	0.018	0.351	0.073	0.132	0.680	0.013	0.160	0.783	0.598	0.012	0.108
**CTSG**	0.524	0.879	0.508	0.038	0.239	0.719	0.839	0.762	0.581	0.280	0.005	0.779	0.016	0.237	0.833	0.103	0.093	0.389	0.511	0.234	0.112
**CXCL8**	0.373	0.710	0.123	0.007	0.437	0.663	0.721	0.685	0.357	0.140	0.020	0.558	0.059	0.236	0.475	0.222	0.106	0.019	0.325	0.921	0.388
**DBT**	0.871	0.240	0.594	0.627	0.311	0.234	0.510	0.179	0.992	0.090	0.259	0.422	0.021	0.238	0.093	0.173	0.601	0.240	0.515	0.163	0.734
**DLAT**	0.817	0.638	0.644	0.494	0.198	0.680	0.355	0.618	0.995	0.620	0.228	0.323	0.354	0.997	0.320	0.040	0.326	0.808	0.417	0.178	0.096
**ECE1**	0.180	0.718	0.808	<0.001	0.213	0.132	0.556	0.808	0.057	0.817	0.011	0.771	0.062	0.075	0.183	0.378	0.023	0.343	0.292	0.806	0.834
**EIF4H**	0.815	0.788	0.714	0.039	0.447	0.690	0.750	0.636	0.225	0.510	0.072	0.920	0.131	0.110	0.854	0.461	0.077	0.981	0.696	0.063	0.106
**ELANE**	0.615	0.518	0.330	0.372	0.693	0.380	0.993	0.691	0.592	0.331	0.029	0.607	0.064	0.418	0.353	0.031	0.184	0.893	0.858	0.185	0.046
**EXOSC10**	0.854	0.325	0.248	0.013	0.676	0.508	0.660	0.920	0.787	0.514	0.547	0.906	0.572	0.572	0.496	0.145	0.348	0.212	0.532	0.106	0.305
**GRP**	0.173	0.203	0.947	0.001	0.194	0.423	0.425	0.157	0.192	0.421	0.378	0.041	0.427	0.443	0.584	0.717	0.213	0.064	0.185	0.614	0.966
**HARS**	0.854	0.577	0.427	0.053	0.301	0.138	0.787	0.782	0.341	0.543	0.047	0.292	0.269	0.269	0.631	0.047	0.210	0.496	0.907	0.007	0.047
**HIST1H4A**	0.748	0.369	0.431	0.003	0.506	0.253	0.591	0.932	0.704	0.218	0.020	0.591	0.024	0.237	0.435	0.192	0.199	0.049	0.567	0.713	0.479
**ICA1**	0.986	0.899	0.117	0.009	0.510	0.283	0.947	0.409	0.648	0.453	0.143	0.311	0.954	0.132	0.926	0.276	0.210	0.178	0.677	0.898	0.122
**IFNA4**	0.845	0.899	0.303	0.150	0.298	0.161	0.978	0.896	0.246	0.552	0.011	0.364	0.032	0.285	0.548	0.014	0.081	0.901	0.346	0.023	0.107
**IFNA6**	0.399	0.597	0.403	0.045	0.432	0.213	0.641	0.853	0.440	0.162	0.010	0.994	0.008	0.214	0.883	0.035	0.103	0.312	0.147	0.666	0.591
**IGF1R**	0.881	0.739	0.544	0.394	0.108	0.454	0.955	0.918	0.479	0.407	0.053	0.247	0.245	0.357	0.593	0.014	0.499	0.664	0.440	0.107	0.201
**IL17A**	0.506	0.621	0.389	0.014	0.054	0.765	0.732	0.882	0.039	0.874	0.067	0.031	0.864	0.070	0.619	0.755	0.134	0.301	0.928	0.453	0.744
**IL4**	0.488	0.793	0.835	0.115	0.066	0.798	0.966	0.279	0.123	0.351	0.016	0.900	0.077	0.304	0.550	0.470	0.015	0.776	0.607	0.477	0.248
**IL6**	0.469	0.716	0.937	0.099	0.719	0.489	0.782	0.028	0.861	0.219	0.096	0.395	0.392	0.430	0.642	0.661	0.154	0.637	0.588	0.014	0.262
**INS**	0.224	0.218	0.482	0.005	0.232	0.795	0.252	0.163	0.679	0.399	0.323	0.792	0.250	0.929	0.742	0.918	0.251	0.620	0.040	0.001	0.072
**LYZ**	0.707	0.843	0.360	0.058	0.205	0.331	0.951	0.776	0.422	0.192	0.001	0.833	0.006	0.120	0.970	0.033	0.032	0.524	0.206	0.172	0.128
**MDA5**	0.444	0.983	0.771	<0.001	0.309	0.741	0.447	0.782	0.252	0.933	0.015	0.143	0.095	0.350	0.379	0.186	0.096	0.005	0.697	0.044	0.038
**MIF**	0.969	0.478	0.640	0.003	0.703	0.344	0.977	0.656	0.489	0.369	0.065	0.229	0.073	0.347	0.900	0.329	0.070	0.008	0.383	0.006	0.001
**MOV10**	0.613	0.859	0.073	0.058	0.019	0.306	0.397	0.918	0.040	0.208	0.015	0.161	0.200	0.216	0.249	0.333	0.079	0.047	0.330	0.113	0.039
**MX1**	0.788	0.993	0.245	0.003	0.712	0.763	0.902	0.099	0.973	0.987	0.027	0.910	0.061	0.306	0.428	0.148	0.119	0.461	0.391	0.016	0.020
**NCL**	0.856	0.437	0.190	0.004	0.292	0.174	0.439	0.853	0.104	0.320	0.020	0.266	0.421	0.059	0.377	0.265	0.279	0.026	0.805	0.126	0.015
**NPM1**	0.661	0.363	0.874	0.026	0.723	0.346	0.457	0.399	0.548	0.632	0.128	0.493	0.122	0.581	0.719	0.036	0.197	0.928	0.940	0.007	0.073
**PRTN3**	0.742	0.557	0.666	0.164	0.917	0.770	0.796	0.101	0.838	0.409	0.013	0.738	0.046	0.284	0.914	0.606	0.014	0.697	0.594	0.246	0.292
**RAE1**	0.466	0.025	0.043	0.507	0.169	0.220	0.325	0.488	0.180	0.450	0.101	0.290	0.094	0.798	0.658	0.500	0.151	0.690	0.197	0.206	0.230
**RNF41**	0.956	0.803	0.736	0.035	0.640	0.992	0.965	0.405	0.478	0.892	0.025	0.809	0.034	0.217	0.353	0.043	0.085	0.669	0.450	0.012	0.024
**RPLP2**	0.952	0.874	0.484	0.146	0.323	0.408	0.894	0.533	0.217	0.484	0.036	0.886	0.068	0.128	0.894	0.045	0.091	0.796	0.900	0.160	0.139
**S100A9**	0.448	0.813	0.065	0.241	0.255	0.560	0.923	0.416	0.231	0.984	0.034	0.508	0.037	0.298	0.567	0.027	0.081	0.696	0.847	0.009	0.093
**SET**	0.899	0.589	0.261	0.226	0.838	0.472	0.155	0.205	0.524	0.428	0.197	0.356	0.102	0.545	0.516	0.018	0.214	0.445	1.000	0.006	0.208
**SLC30A8**	0.169	0.880	0.298	0.569	0.950	0.111	0.573	0.659	0.664	0.158	0.005	0.270	0.020	0.089	0.637	0.154	0.012	0.954	0.831	0.176	0.117
**SNRNP70**	0.281	0.746	0.638	0.007	0.090	0.837	0.663	0.874	0.213	0.486	0.192	0.990	0.144	0.113	0.497	0.071	0.266	0.426	0.697	0.611	0.258
**SNRPA**	0.103	0.932	0.640	0.024	0.699	0.170	0.893	0.680	0.473	0.804	0.544	0.534	0.174	0.526	0.943	0.724	0.611	0.680	0.613	0.508	0.404
**SNRPB**	0.005	0.028	0.353	<0.001	0.498	0.198	0.833	0.133	0.033	0.051	0.093	0.043	0.038	0.060	0.135	0.023	0.085	0.002	0.383	0.877	0.110
**SNRPD1**	0.536	0.945	0.462	0.523	0.319	0.940	0.047	0.755	0.848	0.201	0.519	0.886	0.140	0.820	0.669	0.648	0.225	0.043	0.078	0.066	0.022
**SOX13**	0.399	0.670	0.443	0.616	0.040	0.797	0.669	0.826	0.483	0.603	0.002	0.456	0.089	0.481	0.484	0.539	0.001	0.283	0.888	0.540	0.020
**SRP19**	0.433	0.370	0.086	0.012	0.187	0.047	0.992	0.280	0.110	0.075	0.023	0.478	0.059	0.164	0.746	0.228	0.160	0.202	0.720	0.270	0.033
**SSB**	0.415	0.978	0.897	0.093	0.547	0.851	0.129	0.967	0.961	0.686	0.860	0.722	0.635	0.878	0.573	0.687	0.207	0.016	0.852	0.098	0.027
**TG**	0.747	0.625	0.267	0.313	0.229	0.172	0.985	0.675	0.216	0.777	0.168	0.181	0.417	0.376	0.715	0.036	0.585	0.688	0.607	0.006	0.127
**TGFB1**	0.381	0.181	0.730	0.132	0.751	0.400	0.131	0.307	0.633	0.398	0.876	0.867	0.720	0.356	0.045	0.541	0.483	0.733	0.051	0.008	0.056
**TOP1**	0.980	0.134	0.132	0.007	0.974	0.086	0.958	0.925	0.636	0.095	0.238	0.651	0.505	0.433	0.372	0.295	0.484	0.001	0.815	0.029	0.001
**TRIM21**	0.885	0.028	0.399	0.647	0.775	0.070	0.543	0.447	0.727	0.023	0.176	0.509	0.113	0.698	0.203	0.405	0.450	0.307	0.319	0.241	0.073
**TROVE2**	0.738	0.544	0.129	0.211	0.345	0.167	0.423	0.960	0.410	0.589	0.203	0.196	0.469	0.331	0.502	0.022	0.658	0.791	0.893	0.010	0.108
**UBTF**	0.847	0.517	0.261	0.003	0.908	0.376	0.852	0.954	0.696	0.436	0.050	0.730	0.192	0.262	0.770	0.146	0.097	0.055	0.979	0.059	0.005
**VEGFA**	0.459	0.813	0.119	0.037	0.731	0.543	0.901	0.382	0.470	0.537	0.027	0.968	0.015	0.665	0.811	0.890	0.160	0.319	0.108	0.008	0.001
**VIM**	0.524	0.125	0.761	0.768	0.172	0.622	0.967	0.683	0.630	0.769	0.037	0.469	0.564	0.906	0.536	0.057	0.790	0.348	0.539	0.529	0.942

Table S6. Associations of autoantibody reactivity with symptoms in women. Beta coefficients from age-adjusted regression analysis comparing males with a specific symptom burden to females without the same symptom are shown. The last 3 columns display results from age-adjusted analyses comparing females with different levels of symptoms burden to the pre-pandemic healthy control group.

Beta	Chest pain	Chills	Conjunctivitis	Cough dry	Fever	Loss of appetite	Nasal congestion	Nausea	Shortness of breath	Skin	Smell taste	Sore throat	Vomiting	Asymptomatic	Mild	More than mild
**AQP4**	-0.093	0.029	-0.029	-0.150	0.130	-0.174	-0.251	-0.262	-0.022	-0.252	-0.201	-0.465	0.207	0.479	0.499	-0.121
**C3**	-0.388	-0.248	-0.372	-0.130	0.091	-0.158	-0.138	-0.048	-0.064	0.162	0.177	-0.173	0.470	0.429	0.492	0.274
**CENPB**	-0.065	-0.169	-0.328	-0.239	0.036	-0.141	-0.046	0.069	0.161	0.004	0.204	-0.043	0.700	0.369	0.357	0.443
**CHD3**	-0.385	-0.438	0.019	-0.102	-0.260	-0.117	-0.266	-0.021	-0.007	0.796	0.124	-0.305	0.200	1.041	1.062	0.485
**CHD4**	0.054	-0.404	-0.691	-0.203	-0.116	-0.280	0.120	0.028	0.244	0.153	0.180	-0.156	-0.007	0.111	0.051	0.298
**CHGA**	0.052	-0.149	0.015	-0.003	0.096	-0.118	-0.349	-0.014	0.525	-0.471	-0.046	-0.187	0.062	0.215	0.007	0.068
**DBT**	-0.333	-0.448	-0.426	-0.400	-0.439	0.081	0.096	-0.038	0.001	0.033	0.032	0.055	-0.100	0.740	0.765	0.479
**ECE1**	0.069	-0.178	-0.044	-0.285	0.055	-0.252	0.082	0.054	0.240	-0.288	0.345	-0.125	0.737	0.689	0.478	0.313
**ELANE**	0.351	-0.113	1.044	-0.245	-0.166	-0.502	-0.094	0.140	0.167	-0.065	-0.104	-0.066	0.105	0.548	0.305	0.332
**EXOSC10**	-0.029	-0.222	-0.464	-0.429	-0.187	-0.471	-0.240	-0.031	0.108	0.078	-0.115	-0.150	0.195	0.992	0.372	0.396
**GAD65**	-0.246	-0.193	-0.248	-0.275	-0.098	-0.415	0.011	-0.157	0.090	-0.320	0.129	-0.162	-0.059	0.185	0.211	-0.113
**HARS**	-0.108	-0.215	0.042	-0.197	0.116	-0.059	-0.175	0.085	0.025	-0.313	0.223	-0.178	0.697	0.380	0.416	0.338
**HIST1H4A**	-0.071	-0.129	-0.315	-0.399	-0.088	-0.267	-0.109	-0.019	0.257	0.083	0.052	-0.107	0.326	0.419	0.540	0.399
**IFNA2**	-0.404	-0.199	-0.556	-0.191	0.193	-0.103	-0.238	-0.046	0.058	-0.127	-0.156	0.138	0.632	0.421	0.376	0.042
**IGF1R**	-0.437	-0.124	-0.558	-0.097	0.031	-0.185	-0.218	-0.102	-0.207	-0.179	-0.120	-0.170	0.445	0.471	0.629	0.303
**IL10**	-0.230	-0.330	-0.160	-0.366	-0.155	-0.248	-0.124	-0.011	0.000	0.050	0.001	-0.217	0.247	0.760	0.277	0.293
**INS**	0.310	-0.112	1.450	0.012	-0.211	-0.251	-0.079	-0.002	-0.172	-0.454	0.048	-0.062	0.238	0.442	0.510	0.428
**MOV10**	-0.150	0.022	1.161	-0.025	0.000	-0.024	-0.159	-0.128	-0.208	0.249	0.149	0.069	0.087	-0.195	-0.063	-0.161
**MX1**	0.076	-0.243	-0.525	-0.253	-0.227	-0.500	-0.139	-0.040	0.262	0.079	0.113	-0.104	0.183	0.741	0.524	0.468
**PRTN3**	0.273	-0.201	0.105	-0.144	0.000	-0.475	0.181	-0.227	0.285	0.987	-0.233	-0.340	-0.158	0.384	0.574	0.277
**PTPRN**	0.227	0.072	-0.083	-0.036	0.357	0.024	0.170	0.034	0.299	-0.221	0.311	0.053	0.086	-0.288	0.040	0.151
**RNF41**	-0.140	-0.129	-0.544	-0.233	0.031	-0.423	-0.091	-0.096	0.091	-0.250	0.121	-0.134	0.296	0.677	0.295	0.486
**ROS1**	-0.094	-0.051	-0.314	-0.214	-0.067	-0.371	-0.394	-0.124	-0.144	-0.318	-0.380	-0.262	0.172	0.395	0.203	0.036
**RPLP2**	-0.234	-0.243	-0.269	-0.264	0.048	-0.160	-0.249	-0.044	0.047	-0.221	0.060	-0.053	0.624	0.292	0.447	0.025
**S100A8**	-0.161	0.219	0.547	-0.077	0.383	0.189	-0.129	-0.176	0.142	-0.480	0.063	-0.028	0.289	0.504	0.178	0.472
**S100A9**	-0.355	-0.353	-0.214	-0.335	-0.149	-0.373	-0.286	-0.088	-0.004	-0.236	0.060	-0.298	0.309	1.036	0.544	0.356
**SET**	-0.046	-0.248	-0.482	-0.327	-0.121	-0.527	-0.043	-0.232	-0.029	0.224	-0.254	-0.214	0.424	0.553	0.303	0.038
**SMD3**	0.041	-0.288	-0.175	-0.417	-0.301	-0.269	-0.004	0.027	0.096	0.152	0.270	-0.005	0.259	0.325	0.316	0.287
**SNRPC**	0.124	-0.130	0.071	-0.075	0.148	-0.021	-0.049	0.393	-0.207	-0.196	-0.022	0.237	0.306	0.136	0.185	0.465
**SOX13**	0.088	-0.299	-0.182	-0.155	-0.018	-0.028	0.219	0.039	0.269	-0.701	-0.179	-0.034	-0.475	0.701	1.094	0.693
**SP100**	0.140	0.080	-0.023	-0.015	0.249	-0.102	-0.040	0.016	0.530	-0.203	-0.063	0.208	0.706	0.402	0.172	0.759
**SRP54**	-0.485	-0.188	0.225	-0.084	-0.076	0.120	-0.119	-0.140	-0.130	0.004	-0.005	0.011	-0.322	0.219	0.539	0.185
**TG**	-0.449	-0.256	-0.405	-0.144	0.104	-0.131	-0.251	0.015	-0.117	-0.182	0.151	-0.174	0.527	0.506	0.479	0.268
**TGFB1**	0.258	-0.142	-0.090	-0.298	-0.286	-0.250	-0.068	0.005	0.191	0.072	0.079	-0.382	0.190	-0.077	-0.218	-0.118
**TOP1**	-0.088	-0.200	-0.297	-0.476	-0.183	-0.390	-0.046	-0.001	0.111	0.094	0.179	-0.201	0.302	0.462	0.421	0.344
**TPO**	-0.410	-0.098	-0.621	-0.043	0.174	-0.063	-0.004	-0.434	0.072	0.148	0.036	-0.506	0.128	0.553	0.608	0.159
**TRIM33**	0.024	-0.233	0.267	-0.315	-0.104	-0.177	-0.018	0.098	0.100	0.201	0.004	-0.119	-0.086	-0.004	-0.025	-0.194
**UBTF**	-0.031	-0.221	-0.209	-0.503	-0.209	-0.228	-0.141	0.016	0.090	0.160	0.283	-0.211	0.281	0.580	0.394	0.126

Table S7. Associations of AABs reactivity with symptoms in women. P values from age-adjusted regression analysis comparing males with a specific symptom burden to females without the same symptom are shown. The last 3 columns display results from age-adjusted analyses comparing females with different levels of symptoms burden to the pre-pandemic healthy control group.

P value	Chest pain	Chills	Conjunctivitis	Cough dry	Fever	Loss of appetite	Nasal congestion	Nausea	Shortness of breath	Skin	Smell taste	Sore throat	Vomiting	Asymptomatic	Mild	More than mild
**AQP4**	0.690	0.879	0.951	0.429	0.502	0.363	0.183	0.193	0.918	0.473	0.289	0.014	0.556	0.260	0.154	0.668
**C3**	0.047	0.121	0.346	0.418	0.578	0.327	0.385	0.776	0.714	0.585	0.268	0.285	0.111	0.059	0.075	0.222
**CENPB**	0.773	0.353	0.466	0.189	0.845	0.444	0.800	0.721	0.421	0.991	0.260	0.814	0.036	0.132	0.200	0.096
**CHD3**	0.106	0.023	0.968	0.600	0.190	0.553	0.170	0.921	0.975	0.026	0.525	0.118	0.580	0.003	0.003	0.110
**CHD4**	0.822	0.038	0.152	0.299	0.562	0.156	0.539	0.893	0.256	0.675	0.357	0.430	0.984	0.751	0.882	0.340
**CHGA**	0.822	0.434	0.974	0.989	0.619	0.540	0.064	0.944	0.011	0.179	0.810	0.329	0.859	0.651	0.984	0.810
**DBT**	0.134	0.012	0.340	0.026	0.016	0.660	0.594	0.844	0.996	0.922	0.859	0.762	0.765	0.075	0.012	0.113
**ECE1**	0.739	0.294	0.916	0.090	0.750	0.139	0.629	0.763	0.196	0.358	0.039	0.466	0.017	0.016	0.111	0.184
**ELANE**	0.141	0.563	0.028	0.207	0.402	0.010	0.627	0.500	0.435	0.856	0.592	0.735	0.770	0.044	0.352	0.191
**EXOSC10**	0.906	0.266	0.347	0.030	0.359	0.018	0.227	0.883	0.624	0.834	0.563	0.458	0.598	0.002	0.255	0.184
**GAD65**	0.249	0.267	0.563	0.111	0.579	0.017	0.948	0.395	0.638	0.320	0.456	0.354	0.855	0.632	0.491	0.614
**HARS**	0.637	0.247	0.926	0.290	0.539	0.756	0.346	0.668	0.902	0.363	0.229	0.342	0.041	0.165	0.162	0.230
**HIST1H4A**	0.779	0.526	0.531	0.049	0.672	0.193	0.592	0.931	0.249	0.827	0.797	0.604	0.387	0.176	0.088	0.165
**IFNA2**	0.052	0.243	0.185	0.262	0.265	0.551	0.160	0.799	0.756	0.688	0.358	0.422	0.043	0.099	0.191	0.862
**IGF1R**	0.038	0.472	0.189	0.573	0.860	0.287	0.203	0.579	0.273	0.575	0.485	0.327	0.161	0.139	0.052	0.239
**IL10**	0.295	0.063	0.716	0.039	0.396	0.169	0.487	0.955	1.000	0.879	0.995	0.227	0.455	0.022	0.397	0.288
**INS**	0.221	0.587	0.004	0.954	0.315	0.228	0.702	0.993	0.449	0.234	0.817	0.765	0.533	0.179	0.155	0.123
**MOV10**	0.446	0.890	0.003	0.878	0.999	0.883	0.317	0.452	0.234	0.398	0.350	0.666	0.769	0.585	0.840	0.531
**MX1**	0.750	0.207	0.270	0.188	0.247	0.009	0.469	0.847	0.215	0.826	0.558	0.595	0.608	0.012	0.103	0.069
**PRTN3**	0.271	0.319	0.833	0.476	0.998	0.019	0.369	0.289	0.197	0.007	0.247	0.094	0.673	0.344	0.151	0.366
**PTPRN**	0.298	0.684	0.850	0.837	0.046	0.893	0.334	0.857	0.123	0.501	0.076	0.769	0.793	0.427	0.906	0.615
**RNF41**	0.569	0.518	0.270	0.243	0.878	0.035	0.650	0.651	0.680	0.499	0.544	0.508	0.424	0.034	0.356	0.069
**ROS1**	0.651	0.764	0.449	0.202	0.698	0.027	0.017	0.488	0.434	0.306	0.022	0.121	0.581	0.261	0.507	0.894
**RPLP2**	0.235	0.128	0.497	0.098	0.768	0.323	0.118	0.798	0.789	0.456	0.710	0.746	0.034	0.461	0.184	0.926
**S100A8**	0.475	0.232	0.226	0.677	0.039	0.309	0.483	0.368	0.482	0.157	0.731	0.881	0.395	0.226	0.579	0.116
**S100A9**	0.115	0.054	0.638	0.067	0.426	0.043	0.117	0.655	0.984	0.488	0.745	0.107	0.363	0.001	0.072	0.174
**SET**	0.839	0.177	0.289	0.073	0.518	0.004	0.813	0.236	0.885	0.511	0.165	0.248	0.212	0.178	0.378	0.896
**SMD3**	0.861	0.131	0.712	0.028	0.121	0.163	0.984	0.894	0.646	0.668	0.155	0.979	0.465	0.239	0.312	0.277
**SNRPC**	0.570	0.464	0.872	0.674	0.415	0.907	0.784	0.036	0.289	0.551	0.902	0.185	0.352	0.689	0.540	0.095
**SOX13**	0.693	0.098	0.685	0.392	0.923	0.879	0.224	0.840	0.175	0.035	0.321	0.851	0.155	0.072	0.001	0.014
**SP100**	0.572	0.692	0.964	0.943	0.226	0.618	0.843	0.939	0.016	0.586	0.756	0.307	0.057	0.103	0.532	0.016
**SRP54**	0.018	0.263	0.589	0.620	0.660	0.481	0.480	0.434	0.484	0.990	0.975	0.950	0.302	0.611	0.138	0.517
**TG**	0.031	0.131	0.336	0.398	0.550	0.446	0.138	0.936	0.531	0.564	0.375	0.312	0.093	0.040	0.097	0.274
**TGFB1**	0.231	0.419	0.835	0.088	0.107	0.156	0.698	0.979	0.320	0.825	0.651	0.029	0.559	0.849	0.574	0.689
**TOP1**	0.726	0.326	0.555	0.018	0.379	0.056	0.823	0.998	0.620	0.803	0.380	0.329	0.423	0.150	0.221	0.247
**TPO**	0.081	0.612	0.190	0.822	0.375	0.747	0.984	0.032	0.732	0.677	0.850	0.008	0.720	0.107	0.082	0.579
**TRIM33**	0.902	0.144	0.499	0.047	0.525	0.273	0.912	0.564	0.568	0.497	0.979	0.462	0.771	0.993	0.942	0.487
**UBTF**	0.908	0.303	0.692	0.018	0.337	0.292	0.509	0.944	0.703	0.686	0.185	0.328	0.478	0.057	0.295	0.654

Figure S1. Sex-specific co-occurrence of symptoms. Sex differences in the occurrence of symptoms are visualized using co-occurrence networks, wherein each symptom is denoted with a node and each edge connecting 2 nodes represents the co-occurrences of 2 symptoms that are significantly higher or lower than would be expected by chance using a probabilistic model. Darker solid lines denote co-occurrences with higher frequency.

A. **Co-Occurrence of Symptoms in Men** **B.** **Co-Occurrence of Symptoms in Women**

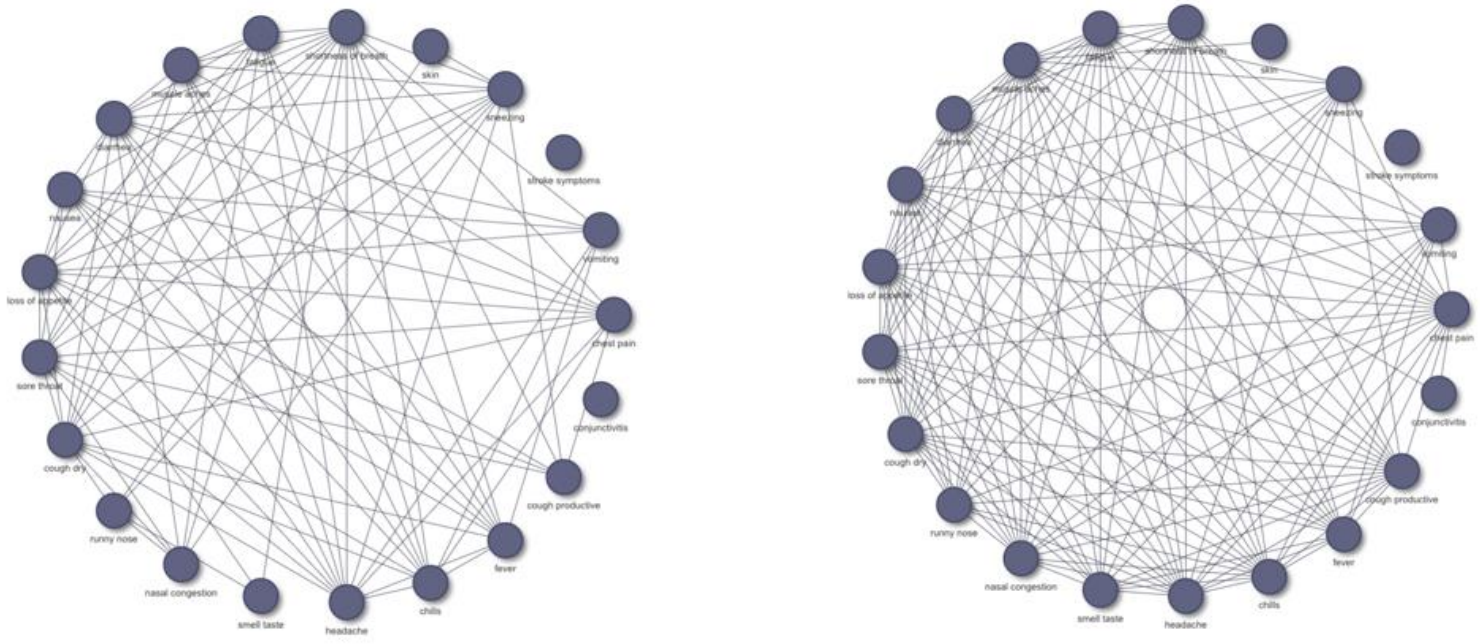

Figure S2. Sex-specific clustering of autoantibody responses overall. Following principal components analyses, K-means clusters of each autoantibody response were derived and plotted in distinct colors (per cluster) along x-axis values of the first principal component (PC) and y-axis values of the second component.

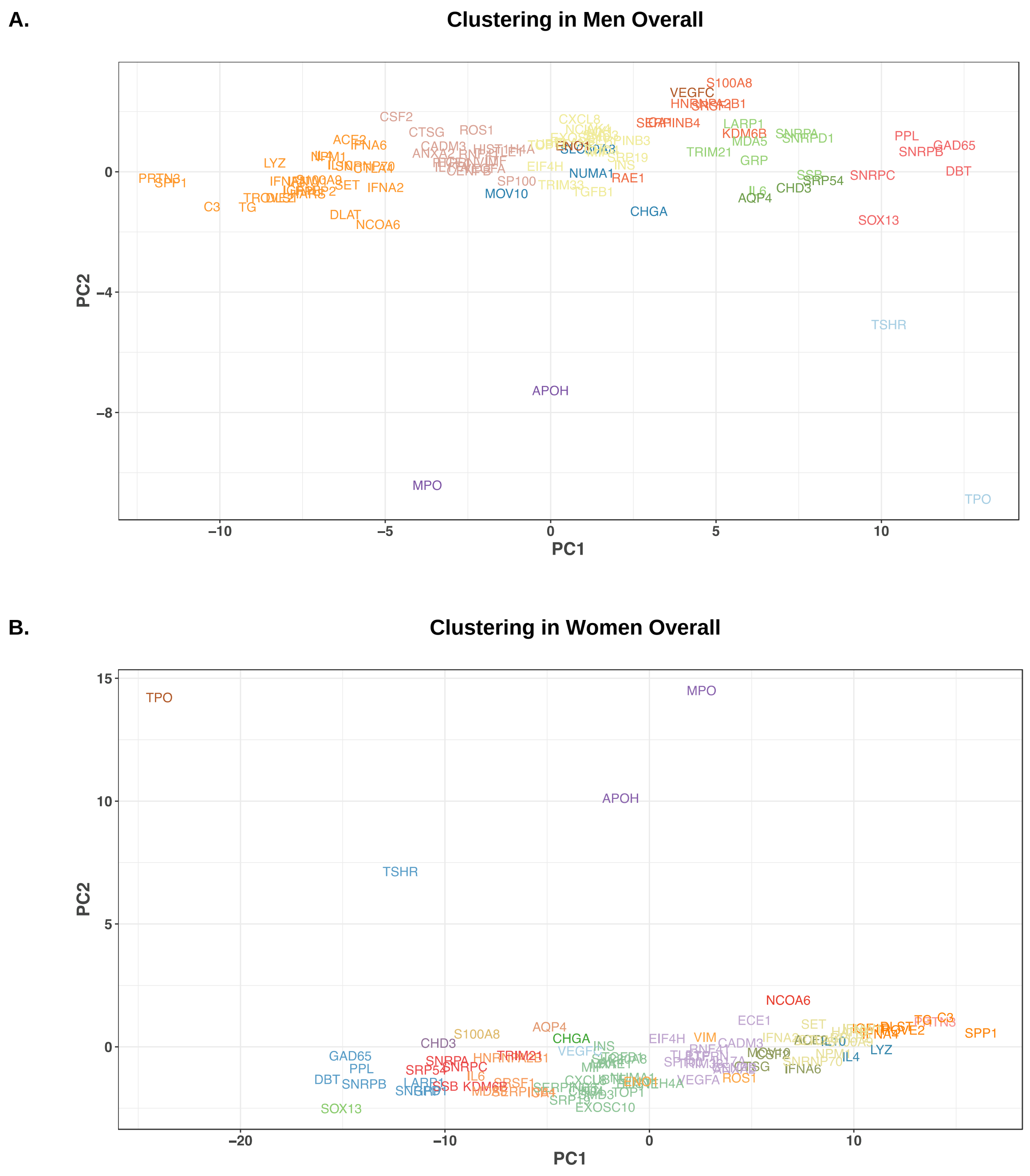

Figure S3. Sex-specific clustering of autoantibody responses by symptom burden. K-means clusters of each autoantibody response adjusted by symptoms burden were derived and plotted in distinct colors (per cluster) along x-axis, the principal component 1 (PC1) and y-axis values of the PC2 of male (A, C, E) and female (B, D, F) HCWs.

A. **Men: Asymptomatic Course**

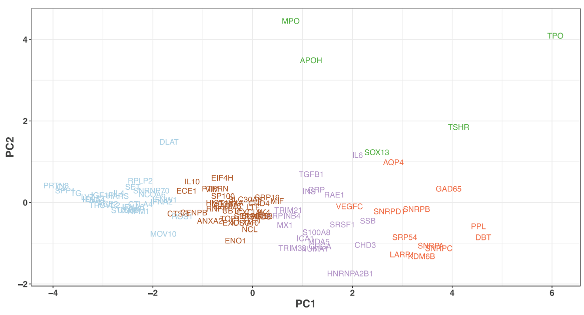

B. **Women: Asymptomatic Course**

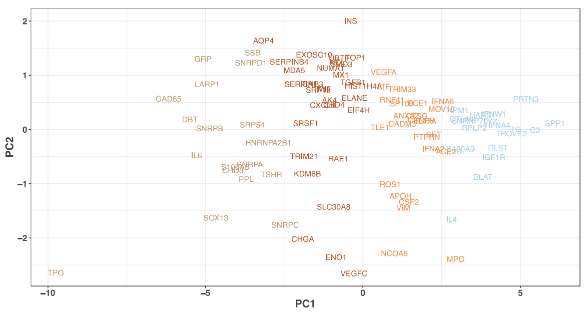

C. **Men: After Mild Symptoms**

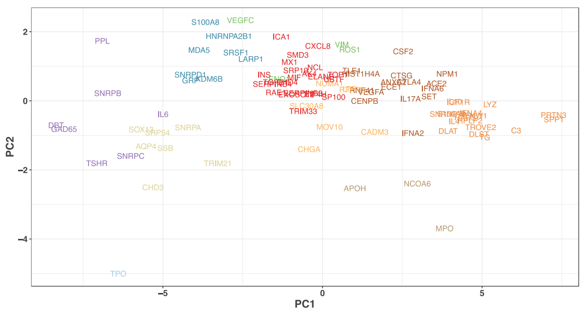

D. **Women: After Mild Symptoms**

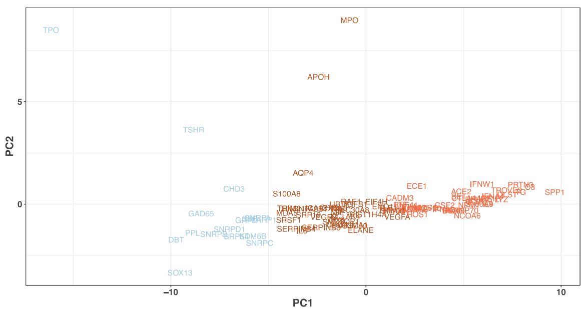

E. **Men: After More than Mild Symptoms**

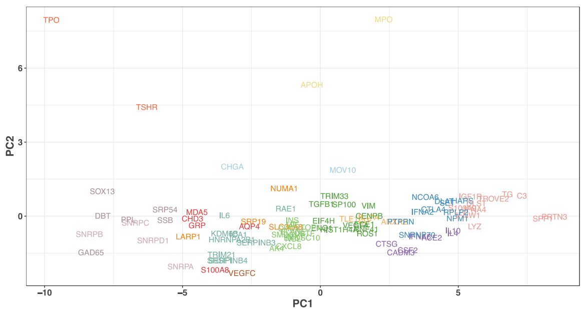

F. **Women: After More than Mild Symptoms**

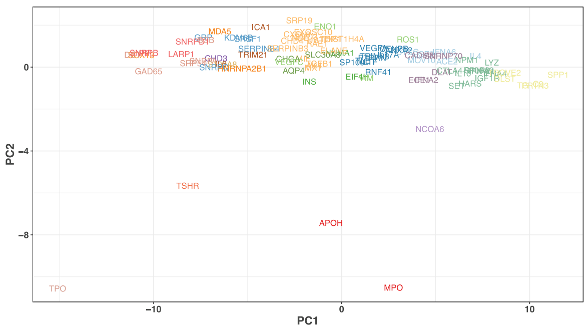

Figure S4. Sex-specific associations of autoantibody reactivity with timing of symptoms, in persons previously infected by SARS-CoV-2. From age-adjusted regression analyses, beta coefficients and negative log p values were obtained from examining the associations of symptoms timing with distinct autoantibodies. A higher symptoms timing score denotes more recent timing, with associations for men in **Panel A** and associations for women in **Panel B**.

A. MEN

B. WOMEN

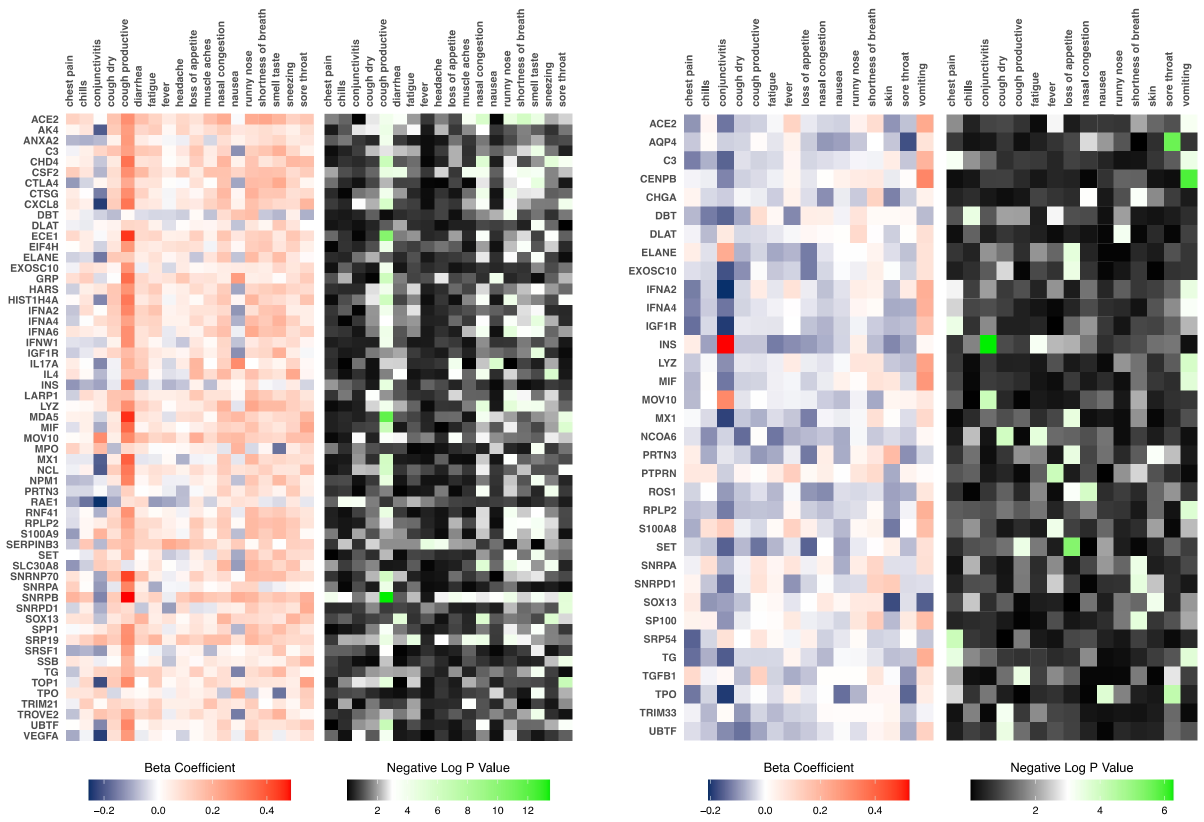

Figure S5. Sex-specific associations of autoantibodies with SLE status. In age-adjusted regression analyses, the breadth and magnitude of associations observed AABs reactivity and systemic lupus erythematosus (SLE) compared to health control status were predominantly seen in women compared to men. Beta coefficients were shown to the left, and negative log P values were shown to the right in each panel.

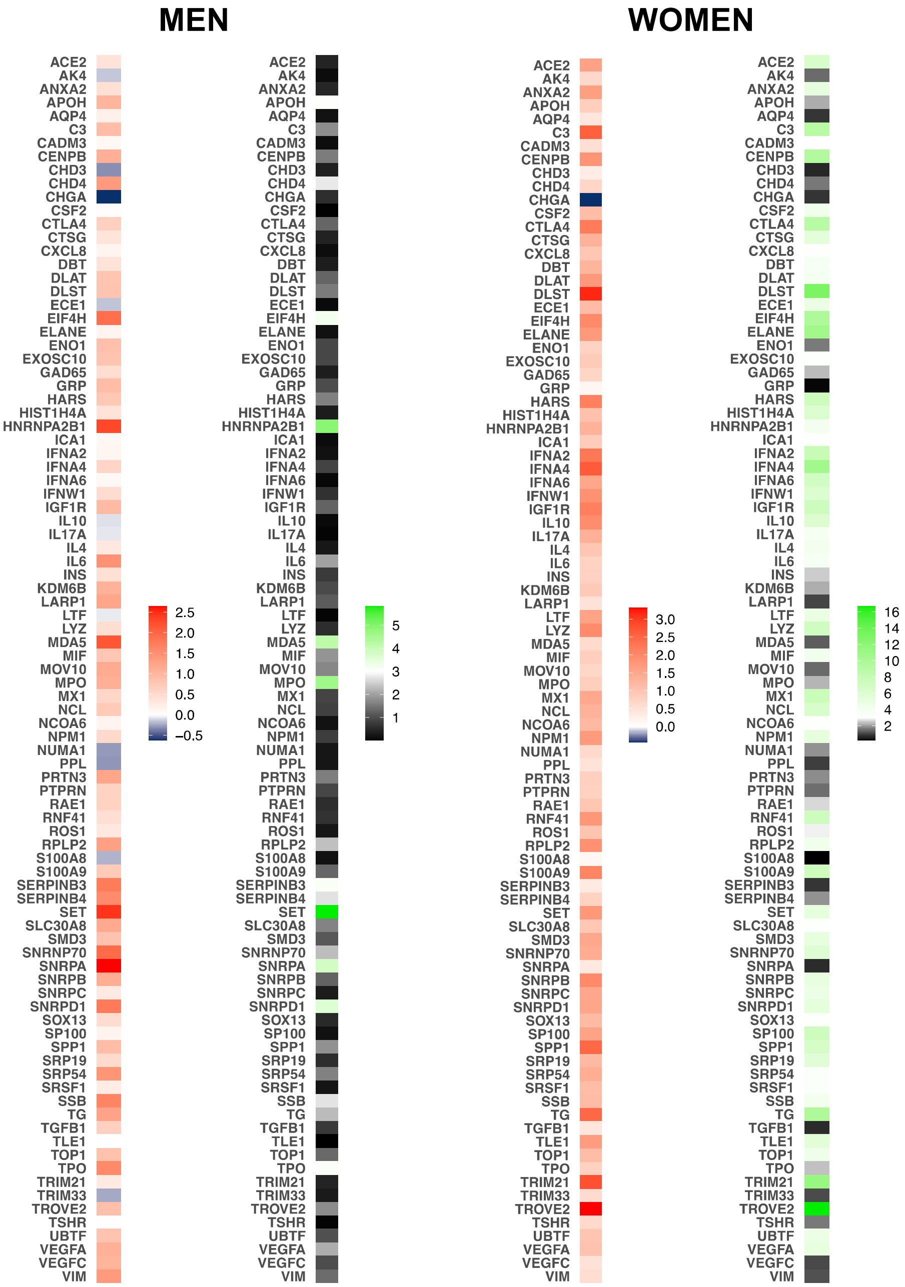

Supplement: Supplementary file 1 — Additional file 1. Supplementary Materials. [file 12967_2021_3184_MOESM1_ESM.pdf]
